# Pallidal Deep Brain Stimulation Enhances Habitual Behavior in a Neuro‐Computational Basal Ganglia Model During a Reward Reversal Learning Task

**DOI:** 10.1111/ejn.70130

**Published:** 2025-05-05

**Authors:** Oliver Maith, Dave Apenburg, Fred Hamker

**Affiliations:** ^1^ Department of Computer Science Chemnitz University of Technology Chemnitz Germany

**Keywords:** decision‐making, human participants, isolated dystonia, rate‐coded, synaptic plasticity

## Abstract

Deep brain stimulation (DBS) within the basal ganglia is a widely used therapeutic intervention for neurological disorders; however, its precise mechanisms of action remain unclear. This study investigates how DBS may affect decision‐making processes through computational modeling of the basal ganglia. A rate‐coded model incorporating direct, indirect, and hyperdirect pathways was utilized alongside a cortico‐thalamic shortcut known for promoting habitual behavior. Simulations of a two‐choice reward reversal learning task were conducted to replicate data from patients with dystonia in ON and OFF DBS conditions. We demonstrate that plasticity in the cortico‐thalamic shortcut, which bypasses the basal ganglia, is crucial for reproducing the patients' behavioral data, emphasizing the role of habit formation. Simulated DBS increased habitual behavior following reward reversal. Integrating different DBS mechanisms revealed that suppression of stimulated neurons, stimulation of efferent axons, and a combined variant promoted habitual behavior. Analyses of thalamic inputs showed that, despite differing effects on the model's activity and plasticity, these DBS variants consistently reduced the influence of the basal ganglia while enhancing the role of the cortico‐thalamic shortcut. Notably, the DBS variants were distinguishable by their divergent behavioral effects following discontinued stimulation. These findings underscore the potential multifaceted effects of DBS on decision‐making processes. In particular, our model proposes that DBS modulates the balance between reward‐guided and habitual behavior.

## Graphical abstract

Using our neuro‐computational model of the basal ganglia, we replicated the behavioral data of dystonia patients in a reversal‐learning task under DBS ON and OFF conditions. Our model suggests that DBS in the globus pallidus internus can diminish the basal ganglia's influence on decision‐making, thereby strengthening cortico‐thalamic connections and therefore promoting habitual behavior. Furthermore, we demonstrate that different DBS mechanisms can result in similar behavioral outcomes while having distinct effects on neuronal activity and plasticity.

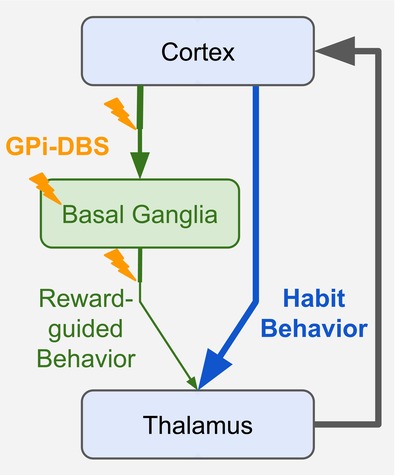

**Abbreviations**
Cordec: cortex population in the model encoding the decision
Corin: cortex population in the model providing input to basal ganglia and thalamus
DBS: deep brain stimulation
DDM: drift diffusion model
exc: excitatory
GPe: globus pallidus externus
GPi: globus pallidus internus
inh: inhibitory
IQR: interquartile range
MCMC: Markov Chain Monte Carlo
PPN: pedunculopontine nucleus
RLDDM: reinforcement learning drift diffusion model
SNc: substantia nigra pars compacta
STN: subthalamic nucleus
StrD1: D1 dopamine receptor‐expressing striatal projection neurons
StrD2: D2 dopamine receptor‐expressing striatal projection neurons
StrThal: thalamic feedback receiving striatal projection neurons
TD: temporal difference (learning model)

## 1 Introduction

Deep brain stimulation (DBS) is a widely used treatment for neurological diseases such as Parkinson's or dystonia especially when conventional medication fails to adequately manage symptoms, particularly motor symptoms (Fan et al. 2021; Hickey and Stacy 2016; Krauss et al. 2021; Lozano et al. 2019; Neumann, Horn, and Kühn 2023). Typically, electrodes deliver high‐frequency current pulses to areas such as the subthalamic nucleus (STN) or globus pallidus internus (GPi) within the basal ganglia. While studies have shown several detailed effects of DBS on nearby tissue, including suppression of local soma (Benazzouz et al. 2000; Boraud et al. 1996; Dostrovsky et al. 2000; Meissner et al. 2005; Shi et al. 2006; Tai et al. 2003; Wu et al. 2001), stimulation of local axons (Anderson et al. 2006; Dvorzhak et al. 2013; Johnson and McIntyre 2008; Kringelbach et al. 2007; Lee et al. 2004; Liu et al. 2008; Miocinovic et al. 2008) also passing fibers (Miocinovic et al. 2006; So et al. 2012), the exact mechanisms affecting basal ganglia function remain unclear. One prevailing hypothesis is that DBS may cause a functional lesion in the stimulated region (Neumann, Horn, and Kühn 2023).

The basal ganglia are a group of subcortical structures that play a pivotal role in motor control, and various aspects of behavior such as decision‐making, reward processing, reward‐based learning, goal‐directed, and habitual behavior (Cui et al. 2013; DeLong and Wichmann 2009; Humphries and Prescott 2010; Mink 1996; Nelson and Kreitzer 2014; Schroll and Hamker 2013; Schultz 2016; Yin and Knowlton 2006). A widely used model divides the basal ganglia into direct and indirect pathways (Alexander and Crutcher 1990; Baladron and Hamker 2015; DeLong 1990; Frank 2005; Gurney et al. 2001; Schroll et al. 2014). Activation of the former reduces the ongoing inhibition from the GPi toward its target structures, facilitating motor actions or decision‐making. Conversely, activation of the indirect pathway produces the opposite effect. Dopamine‐modulated synaptic plasticity regulates both pathways (Schroll et al. 2014; Schultz et al. 2003; Shen et al. 2008), where transient dopamine changes encode a reward prediction error (Diederen and Fletcher 2021; Schultz et al. 1997). Therefore, it is hypothesized that the direct pathway facilitates rewarded actions, while the indirect pathway suppresses unrewarded ones, even though both are concurrently active during action initiation (Cui et al. 2013).

Computational modeling serves as an effective tool for exploring the functional effects of DBS on the basal ganglia, allowing for the testing of diverse mechanisms without invasive interventions in patients or animals. It has already contributed significantly to the understanding of the basal ganglia and DBS (Feng et al. 2007; Frank et al. 2007; Hahn and McIntyre 2010; Holt et al. 2016; Holt and Netoff 2014; Kumar et al. 2011; Neumann et al. 2018; Rubin and Terman 2004; So et al. 2012).

The basal ganglia are central to regulating habitual and goal‐directed behavior. It is proposed that the dorsomedial striatum supports goal‐directed control, while the dorsolateral striatum underpins habits, with Parkinson's disease impairing habitual effects on behavior due to dopamine loss in sensorimotor regions (Redgrave et al. 2010). Baladron and Hamker (2020) extended this framework, proposing a hierarchical organization of cortico‐basal ganglia loops, where habits form as cortico‐thalamic and cortico‐cortical shortcuts bypassing goal‐directed loops. Consistent with this, Smith and Graybiel (2013) demonstrated that during overtraining, the sensorimotor striatum and infralimbic cortex develop habit‐related activity. Notably, the infralimbic cortex exhibits this activity later than the striatum, and optogenetic disruption of infralimbic activity during overtraining prevented habit formation, suggesting it participates in a slower learning process forming habits, potentially reflecting cortico‐cortical shortcuts facilitated by basal ganglia training. In our recent neuro‐computational modeling studies, we investigated how the basal ganglia can guide the training of such slow‐learning shortcuts through reward‐based learning (Baladron and Hamker 2020; Scholl et al. 2022; Schroll et al. 2014; Villagrasa et al. 2018).

Within the framework of cortico‐thalamic and cortico‐cortical shortcuts bypassing the basal ganglia, both the basal ganglia and these slow‐learning shortcut connections contribute to decision‐making. This raises a critical question: To what extent do the basal ganglia influence behavior compared with these shortcut connections, and what mechanisms regulate this balance? In this work, we propose that DBS within the basal ganglia could modulate this balance, thereby regulating the interplay between reward‐guided and habitual behavior.

We hypothesize that DBS, particularly within the GPi, diminishes the influence of the basal ganglia on decision‐making, thereby amplifying the influence of cortico‐thalamic shortcuts. This perspective aligns with the informational lesion hypothesis regarding the functional effects of DBS (Grill et al. 2004; Neumann, Horn, and Kühn 2023). In this respect, studies have shown that DBS not only suppresses the local soma but also decouples the soma and axons by stimulating local axons (Anderson et al. 2003; Anderson et al. 2018; Hashimoto et al. 2003; McIntyre et al. 2004). Given that the cortico‐thalamic shortcut biases decision‐making toward habitual behavior, our model predicts that DBS within the GPi favors habitual behavior.

To test this hypothesis, we use a basal ganglia model including a cortico‐thalamic shortcut bypassing the basal ganglia circuit to simulate a version of a two‐choice reward reversal learning task based on the study of de A Marcelino et al. (2023). Their study, conducted with dystonia patients, revealed a tendency for increased unrewarded decisions following reward reversal under DBS. Patients also exhibited a prolonged increase in unrewarded decisions compared with the initial learning phase. We propose, according to our basal ganglia model, that these findings can be explained by a habitual bias which is further enhanced due to DBS within the GPi.

de A Marcelino et al. (2023) interpreted their results in the context of an exploration‐exploitation tradeoff, using a temporal difference (TD) learning model. They found that DBS within the GPi increased the proportion of low‐value decisions, as determined by the TD‐learned values. In the framework of epsilon‐greedy or softmax decision rules, such decisions are classified as exploratory (Sutton and Barto 2018). Based on this, de A Marcelino et al. (2023) proposed that DBS within the GPi promotes exploration by functionally disinhibiting GPi target structures.

In this study, we discuss whether the observed behavior truly reflects increased exploration or an enhanced habitual bias. We argue that low‐value choices in a two‐choice reward reversal learning task are not necessarily an indication of exploratory behavior, but may rather indicate a habitual bias. This compelling alternative interpretation grounded in the mechanisms of our neuro‐computational model is supported by applying the TD learning analysis, previously applied to patient data, to our neuro‐computational model's behavior. This analysis shows that even though our model implements a habitual bias by learning shortcuts it similarly produces an increased frequency of low‐value decisions following DBS in the GPi.

Habits are typically assessed using reward devaluation tasks rather than reversal learning tasks (de Wit et al. 2018). By definition, habits are behaviors that persist despite changes in outcomes, precluding relearning. Using our neuro‐computational models, we have recently demonstrated that such outcome‐insensitive habits can emerge after overtraining via cortico‐thalamic and cortico‐cortical shortcuts (Baladron and Hamker 2020; Scholl et al. 2022). However, in the reversal learning task used here, overtraining does not occur, and relearning remains possible. Crucially, our models incorporate the gradual development of habits through slow‐learning plastic connections, leading to these shortcuts gaining influence over time. Thus, in the present study, we refer to habitual behavior as habitual biases that shape behavior while remaining adaptive and outcome‐sensitive.

To identify which effects of DBS on nearby tissue lead to behavioral changes similar to those observed in patients in the study by de A Marcelino et al. (2023), we implement the known effects separately and a combination of them into our model. Unlike previous basal ganglia models comparable with ours, which mainly modeled DBS by a simple input current (Feng et al. 2007; Frank et al. 2007; Rubin and Terman 2004; Su et al. 2018), we aim to bridge the gap between these effects on the nearby tissue and their still unclear functional consequences within the basal ganglia circuit.

## 2 Materials and Methods

### 2.1 Model Overview

The rate‐coded basal ganglia model used in this study builds on our previous work (Maith, Schwarz, and Hamker 2021; Schroll et al. 2014; Villagrasa et al. 2018) and is implemented in Python using the ANNarchy neurosimulator version 4.7.3b (Vitay et al. 2015). Each region within the basal ganglia, along with its input and output regions, is represented by distinct neuron populations connected through projections. The dynamics of these populations and projections are defined by their specific neuron and synapse types (see Sections 2.2 and 2.3).

**FIGURE 1:**
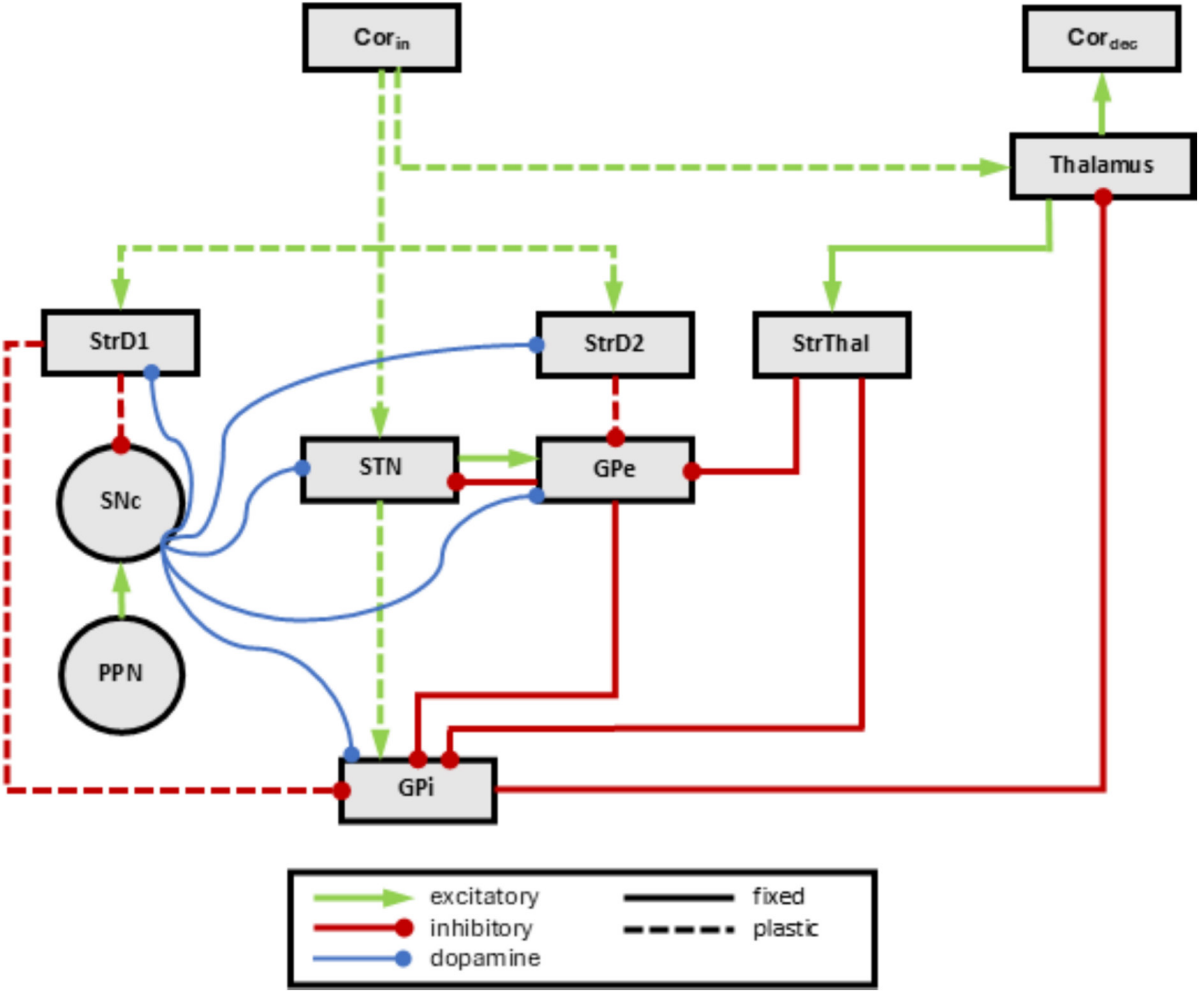
Model overview, rectangles represent neuron populations, circles single neurons, and arrows projections. For simplicity, local projections in StrD1, StrD2, StrThal, STN, and GPi implementing local competition are not shown. The cortex consists of two populations providing input to the basal ganglia and thalamus (Cor_in_) and encoding the decision of the model (Cor_dec_). Details of the populations and projections are shown in Table 1 and Table 2. GPe – globus pallidus externus; GPi – globus pallidus internus; PPN – pedunculopontine nucleus; SNc – substantia nigra pars compacta; STN – subthalamic nucleus; StrD1 – D1 dopamine receptor‐expressing striatal projection neurons; StrD2 – D2 dopamine receptor‐expressing striatal projection neurons; StrThal – thalamic feedback receiving striatal projection neurons.

The striatum is divided into three populations: StrD1 (neurons predominantly with D1‐type dopamine receptors), StrD2 (neurons predominantly with D2‐type dopamine receptors), and StrThal (neurons receiving thalamic feedback instead of cortical input) (Gerfen et al. 1990; Surmeier et al. 2007). The thalamic feedback addresses the credit‐assignment problem in our model by relaying information about action selection back into the basal ganglia, as proposed by Brown et al. (2004). This represents a simplified representation of the diverse neuron types and integrated inputs received by different neurons in the striatum (Hjorth et al. 2020; Johansson and Silberberg 2020). Other structures are represented by individual populations (see Figure 1).

Consistent with our recent studies (Baladron and Hamker 2020; Goenner et al. 2021; Maith, Schwarz, and Hamker 2021; Maith, Villagrasa, Dinkelbach, 2021; Scholl et al. 2022; Villagrasa et al. 2018), the basal ganglia model is organized into three pathways all activated by the cortex (Cor_in_) and converging in the globus pallidus internus (GPi): the direct pathway (Cor_in_‐StrD1‐GPi), the indirect pathway (Cor_in_‐StrD2‐GPe‐GPi), and the hyperdirect pathway (Cor_in_‐STN‐GPi) (Albin et al. 1989; DeLong 1990; Kita et al. 2006; Mink 1996; Nambu et al. 2002; Schroll and Hamker 2013). The converging activity of these pathways, driven by Cor_in_ activation, results in the suppression of a GPi neuron and the subsequent disinhibition of the associated thalamic and cortex neuron (Cor_dec_). Decision‐making in the model is based on the activity within a separate cortical population, Cor_dec_. Thus, our model represents a cortico‐basal ganglia‐thalamo‐cortical loop, which could be mirrored in the brain in various ways (Alexander et al. 1986; Baladron and Hamker 2020; Haber 2003; Trapp et al. 2012). While we refrain from committing to specific cortical regions, a plausible candidate could be an open visual cortico‐striatal loop, potentially involving the inferior temporal and prefrontal cortices (Seger 2013).

To support clear decision‐making and learning in the basal ganglia, lateral competition is integrated into the input regions (StrD1, StrD2, StrThal, and STN) and the GPi. In the model, this competition is represented in a simplified manner through direct inhibitory connections between neurons, abstracting the complex local circuits that may underlie such competition (Brown et al. 2014; Burke et al. 2017; Hjorth et al. 2020; Silberberg and Bolam 2015).

The function of the basal ganglia pathways in a particular task is not pre‐determined by their connectivity but instead emerges through synaptic plasticity. Consistent with empirical findings (Gerfen et al. 1990; Shen et al. 2008), we assume that synaptic plasticity is governed by the interaction of dopamine, presynaptic activity, and postsynaptic activity (Schroll et al. 2014). Phasic dopamine level changes, represented by activity changes in the substantia nigra pars compacta (SNc), reflect reward prediction errors (Diederen and Fletcher 2021; Schultz et al. 1997). Reward prediction is modeled as a plastic inhibitory projection originating from StrD1 neurons. This represents a highly simplified depiction of the complex dopaminergic system in the basal ganglia (Vitay and Hamker 2014), with the inhibitory reward‐predicting projection from the striatum likely originating primarily from the ventral striatum (Humphries and Prescott 2010). The resulting behavioral effects of the implemented plasticity are: In the direct pathway, dopamine‐modulated plasticity enhances the probability of re‐selecting rewarded decisions. The indirect pathway learns to suppress nonrewarded decisions, while the hyperdirect pathway learns to enable rewarded decisions and suppress alternatives. Detailed implementation of the synaptic plasticity within the basal ganglia is provided in Section S1.

Additionally, this model incorporates a shortcut pathway from the cortex (Cor_in_) to the thalamus, bypassing the basal ganglia. This shortcut pathway undergoes slow Hebbian‐like synaptic plasticity. Our previous studies demonstrated that this shortcut could be trained by the fast, reward‐based learning of the basal ganglia, leading to stable input‐decision associations and the formation of habitual behaviors (Baladron and Hamker 2020; Scholl et al. 2022; Schroll et al. 2014; Villagrasa et al. 2018).

### 2.2 Neurons

All neuron populations in this study are characterized by specific artificial rate‐coded neuron types, whose membrane potential is governed by a time‐dependent differential equation and transformed into a mean firing rate through a nonlinear transfer function (Vitay and Hamker 2010). Time‐dependent variables are updated with a time step of 1 ms, and differential equations are solved using the forward Euler method.

The neurons of all populations, except the SNc, are described by the following equations:

(1.1) $$\tau \frac{\mathrm{dmp}}{\mathrm{dt}}=-\mathrm{mp}+B+\sum {\mathrm{psp}}_{\mathrm{exc}}-\sum {\mathrm{psp}}_{\mathrm{inh}}+\lambda \;\text{Uniform}\left(-1,1\right)$$

(1.2) $$r={\left[\mathrm{mp}\right]}^{+}$$

(1.3) $${\left[x\right]}^{+}=\left\{\begin{array}{c} 0,\;x<0\\ x,\;x\geq 0 \end{array}\right.$$

where mp is the membrane potential, r is the neuronal firing rate, τ is the time constant, B is the baseline input, $\sum {\mathrm{psp}}_{\mathrm{exc}}$ is the summed excitatory input, $\sum {\mathrm{psp}}_{\mathrm{inh}}$ is the summed inhibitory input, and λ is the noise amplitude. $\text{Uniform}\left(-1,1\right)$ represents a random number drawn from a uniform distribution every timestep, with −1 and 1 as lower and upper bound. The activity of these populations, except the Cor_in_ and pedunculopontine nucleus (PPN), is regulated by their respective excitatory and inhibitory inputs without manual intervention during simulation. The neurons of Cor_in_ and PPN do not contain synaptic input or noise, and their baseline B is manually set to 1 to simulate the presentation of an input stimulus or reward and is otherwise 0.

The time constant τ is 1 ms for PPN and 10 ms for all other populations. All other parameters for all populations are provided in Table 1. The parameter values are based on our previous modeling study (Maith, Schwarz, and Hamker 2021), with adjustments made to account for the task's different action space, which involves only two possible decisions and the different input encoding (see Section S2).

**TABLE 1:** Population parameters.

Population	Number of neurons	B	λ
Cor_in_	2	—	—
PPN	1	—	—
SNc	1	0.1	—
StrD1	4	0.1	0.1
StrD2	4	0.1	0.1
STN	4	0.1	0.1
GPi	2	2.1	0.1
GPe	2	1.0	0.1
Thalamus	2	1.0	0.0001
Cor_dec_	2	0.0	0.05
StrThal	2	0.25	0.1

*Note:* Baseline B of Cor_in_ and PPN is manually set to 0 or 1 during simulation.

The SNc is modeled using the following equations:

(2.1) $$\tau \frac{\mathrm{dmp}}{\mathrm{dt}}=-\mathrm{mp}+\alpha \;\delta +B$$

(2.2) $$\begin{array}{c} \delta =\left\{\begin{array}{c} {\left[1-B-\sum {\mathrm{psp}}_{\text{StrD}1}\right]}^{+},\;{\mathrm{psp}}_{\mathrm{PPN}}>0\\ \;-10\sum {\mathrm{psp}}_{\text{StrD}1},\;{\mathrm{psp}}_{\mathrm{PPN}}\leq 0 \end{array}\right. \end{array}$$

(2.3) $$r={\left[\mathrm{mp}\right]}^{+}$$

where mp is the membrane potential, r is the neuronal rate, τ is the time constant, B is the baseline input, ${\mathrm{psp}}_{\mathrm{PPN}}$ is the input from the PPN neuron, $\sum {\mathrm{psp}}_{\text{StrD}1}$ is the summed input from the StrD1, and δ is the reward prediction error. The parameter α is set to 1 to allow for a phasic change in SNc activity during reward presentation and is 0 otherwise.

The signal transmitted from a presynaptic neuron to a postsynaptic neuron, referred to as the postsynaptic potential (psp), is defined by the following equation:

(3.1) $$\mathrm{psp}=w\;r_{\mathrm{pre}}$$

Here, w represents the synaptic weight and $r_{\mathrm{pre}}$ denotes the presynaptic neuron's firing rate at the current time step. The postsynaptic potentials from all incoming excitatory synapses contribute to the total excitatory postsynaptic potential, $\sum {\mathrm{psp}}_{\mathrm{exc}}$, while the postsynaptic potentials from all inhibitory synapses contribute to the total inhibitory postsynaptic potential, $\sum {\mathrm{psp}}_{\mathrm{inh}}$ (see Equation 1.1).

### 2.3 Synapses

The populations are interconnected by projections consisting of either fixed, nonlearning synapses or plastic, learning synapses. The connectivity patterns between neurons can be either one‐to‐one, where each presynaptic neuron connects to a single postsynaptic neuron, or all‐to‐all, where each presynaptic neuron connects to every postsynaptic neuron. The connection types and the initial synaptic weights of the projections of the model are provided in Table 2. As for the neuron parameters, the parameter values are based on our previous modeling study (Maith, Schwarz, and Hamker 2021), with slight adjustments made (see Section S2). The plastic connections are initialized using a uniform distribution to introduce heterogeneity across simulations. The plastic shortcut, however, is assigned an initial weight of 0.1 to match the fixed shortcut. Additionally, the plastic connection from StrD2 to GPe is initialized at 0, as it would decrease to 0 rapidly at the beginning of the simulations regardless.

**TABLE 2:** Connection types and weights of the model projections.

Presynaptic	Postsynaptic	Connection	w	Target
GPe	GPi	one_to_one	1.5	inh
GPe	STN	all_to_all	0.1	inh
GPi	GPi	all_to_all	0.9	inh
GPi	Thalamus	one_to_one	1.0	inh
Cor_in_	STN*	all_to_all	(0, 0.1)	exc
Cor_in_	StrD1*	all_to_all	(0, 0.1)	exc
Cor_in_	StrD2*	all_to_all	(0, 0.1)	exc
Cor_in_	Thalamus	all_to_all	0.1	exc
Cor_in_	Thalamus*	all_to_all	0.1	exc
PPN	SNc	all_to_all	1.0	exc
STN	Gpe	all_to_all	0.1	exc
STN	GPi*	all_to_all	(0, 0.05)	exc
STN	STN	all_to_all	1.0	inh
StrD1	GPi*	all_to_all	(0, 0.05)	inh
StrD1	SNc*	all_to_all	0.1	inh
StrD1	StrD1	all_to_all	1.0	inh
StrD2	Gpe*	all_to_all	0.0	inh
StrD2	StrD2	all_to_all	1.2	inh
StrThal	GPe	one_to_one	0.3	inh
StrThal	GPi	one_to_one	0.5	inh
StrThal	StrThal	all_to_all	1.0	inh
Thalamus	Cor_dec_	one_to_one	1.0	exc
Thalamus	StrThal	one_to_one	0.5	exc

*Note:* Asterisk (*) indicates plastic projections whose weights change during simulation. Two values in parentheses in the weight column represent the lower and upper bounds of a uniform distribution from which the weights for each synapse are drawn.

Plastic synapses in the shortcut projection from the Cor_in_ to the thalamus follow a learning rule based on a combination of Hebbian, Oja's, and covariance learning principles, as expressed in the following equation:

(4.1) $$\tau_{w}\frac{\mathrm{dw}}{\mathrm{dt}}=r_{\mathrm{pre}}v-\alpha \;v^{2}w$$

(4.2) $$\alpha =\beta_{\mathrm{reg}}{\left(r_{\text{post}}-\theta_{\mathrm{reg}}\right)}^{+}$$

(4.3) $$v={\left(r_{\text{post}}-{\bar{r}}_{\text{post}}-\theta_{\text{post}}\right)}^{+}$$

This rule involves the time constants $\tau_{w}$, the presynaptic and postsynaptic rates $r_{\mathrm{pre}}$ and $r_{\text{post}}$, the population average of the postsynaptic rate ${\bar{r}}_{\text{post}}$, the postsynaptic rate threshold $\theta_{\text{post}}$, the regularization threshold $\theta_{\mathrm{reg}}$, and a regularization scaling factor $\beta_{\mathrm{reg}}$. The regularization scaling factor $\beta_{\mathrm{reg}}$ ensures that the regularization term in Equation (4.1) becomes active only when thalamic activity exceeds a certain threshold $\theta_{\mathrm{reg}}$, preventing regularization driven solely by thalamic baseline activity. The parameters specific to the plasticity of the shortcut projection are detailed in Table 3.

**TABLE 3:** Cortico‐thalamic projection's plasticity parameters.

Parameter	Value
$\tau_{w}$	150,000
$\theta_{\mathrm{reg}}$	0.93
$\theta_{\text{post}}$	0.1
$\beta_{\mathrm{reg}}$	2.0

The equations and parameters governing the learning rules of the dopamine‐modulated projections in the basal ganglia are provided in Section S1.

### 2.4 Simulating the Task

We initially conducted 14 simulations of the two‐choice reward reversal learning task based on the study by de A Marcelino et al. (2023) to replicate the dystonia patients' behavior data. We did not explicitly incorporate dystonia status into our model, as dystonia primarily causes motor impairments (Bhatia et al. 2018), while our model focuses solely on decision‐making data and does not explicitly represent the motor basal ganglia loop. We subsequently increased our sample size to 100 by running additional simulations. Each simulation was initialized with a different seed for random number generation, which determined the initial weight values (see Table 2), neuronal noise (see Equation 1.1), and random reward distributions.

In line with the study by de A Marcelino et al. (2023), each task run (i.e., simulation with the model) consists of 120 trials, divided into three sessions of 40 trials each for most analyses. During each trial, patients or the model must select one of two fractals, followed by receiving a reward. Based on the reward feedback, the patients and the model are expected to learn to choose the option with a higher reward frequency. Unbeknownst to the patients and the model, the reward probabilities for the two fractals are initially set to 80:20 and are reversed to 20:80 after 60 trials. Each patient performs the task twice, once in the DBS OFF state and once in the DBS ON state, and similarly, each simulation is run for each DBS condition (*DBS OFF*, *suppression*, *afferent*, *efferent*, *passing fibers*, *combined*).

At the start of each trial, a 100 ms period is simulated without input. Following this, inputs to the cortex (B) are set, simulating the presentation of two fractals shown to the human patients. In our model simulations, the position of the fractals is irrelevant and thus not included in the input. In the experimental study, patients had to select the fractals by choosing a side (left/right), necessitating the randomizing of the fractal positions. In contrast, our model directly selects the fractals. A decision by the model is made when the firing rate r of a Cor_dec_ neuron exceeds a threshold value of 0.8 or when a maximum duration of 3000 ms is reached. The fractal corresponding to the Cor_dec_ neuron with the highest activity is chosen.

Upon selecting the fractal with an 80% reward probability, the baseline activity B of the PPN is adjusted to 1 (80% of the time) or to 0 (20% of the time) for 60 ms, and the parameter α of the SNc is set to 1, allowing for phasic dopamine changes. This setup should allow the model to learn to preferentially select the more frequently rewarded fractal.

### 2.5 Integration of DBS

The different models of DBS effects are implemented using the Python package CompNeuroPy version 1.0.1 (Maith 2024), which supports DBS implementation for both rate‐coded and spiking ANNarchy models. First, a region to be stimulated is defined. In this study, the GPi population was selected for stimulation, consistent with the DBS target in the study of de A Marcelino et al. (2023). The following effects can be implemented for rate‐coded models: suppression of neurons in the stimulated region, stimulation of afferent and efferent axons of neurons in the stimulated region, and stimulation of axons in passing fibers. Each effect can be adjusted using specific parameters. In this work, the projection from GPe to STN was selected as a passing fiber (So et al. 2012).

For suppressing local neurons, the membrane potential equation (Equation 1.1) is extended by an inhibitory current, as described by this equation:

(5.1) $$\tau \frac{\mathrm{dmp}}{\mathrm{dt}}=\ldots +\alpha_{\text{suppress}}\;\mathrm{dbs}\;{\left[-1-\mathrm{mp}\right]}_{-}$$

(5.2) $${\left[x\right]}_{-}=\left\{\begin{array}{c} x,\;x<0\\ 0,\;x\geq 0 \end{array}\right.$$

The parameter $\alpha_{\text{suppress}}$ regulates the strength of the suppression, and the parameter dbs is set to 1 to apply DBS and 0 otherwise.

The stimulation of axons is implemented to simulate the effect of DBS to elicit action potentials independently of soma activity. This is implemented in the rate‐coded model with an additional rate $r_{\text{axon}}$ for neurons whose axons are stimulated:

(6.1) $$r_{\text{axon}}=\alpha_{\text{axon}}\;\mathrm{dbs}$$

The parameter $\alpha_{\text{axon}}$ defines this rate for a population. In the synaptic equation, the presynaptic neuron rate $r_{\mathrm{pre}}$ is replaced by the sum of $r_{\mathrm{pre}}$ and $r_{\text{axon}}$:

(7.1) $$r_{\mathrm{pre}}=r_{\mathrm{pre}}+r_{\text{axon}}$$

We here investigate five different DBS variants: exclusive suppression of local neurons (*suppression*), exclusive stimulation of afferent axons (*afferent*), exclusive stimulation of efferent axons (*efferent*), exclusive stimulation of the axons in the projection from GPe to STN (*passing fibers*), and a combined variant (*combined*). The parameters of the different variants are shown in Table 4. How the parameters were obtained is described in Section 3.8.

**TABLE 4:** DBS variants' parameters.

DBS variant	$\alpha_{\text{suppress}}$	$\alpha_{\text{axon}}$ afferents	$\alpha_{\text{axon}}$ efferents	$\alpha_{\text{axon}}$ GPe‐STN
*Suppression*	0.1	0	0	0
*Afferent*	0	0.15	0	0
*Efferent*	0	0	0.05	0
*Passing fibers*	0	0	0	0.38
*Combined*	0.1	0	0.03	0.23

*Note:* $\alpha_{\text{suppress}}$ is set for the neurons of GPi. $\alpha_{\text{axon}}$ is set for projections (either afferents or efferents of GPi or the GPe‐STN projection).

### 2.6 Temporal Difference Learning Model

Using the temporal difference (TD) learning model, we calculated the likelihood of each choice using a Bernoulli distribution:

(8.1) $$A\sim B\left(p\right)$$

where A represents the two possible choices (1 for choice 1 and 0 for choice 2) and p the probability of selecting choice 1. This probability is derived from the *Q*‐values of the TD model as follows:

(9.1) $$p=P\left(A=1\right)=\frac{e^{\beta Q_{1}}}{e^{\beta Q_{1}}+e^{\beta Q_{2}}}$$

Here, β is the scaling parameter of the softmax rule used for action selection, and $Q_{1}$ and $Q_{2}$ are the *Q*‐values of choice 1 and 2, respectively. The *Q*‐value of a choice is updated throughout the task using the TD learning rule:

(10.1) $$Q_{a}\leftarrow Q_{a}+\alpha \left(r_{t}-Q_{a}\right)$$

where α is the learning rate, and $r_{t}$ represents the outcome of the choice on trial t.

As described by de A Marcelino et al. (2023), we fitted two variations of the TD learning model. The first variation used a single learning rate, while the second used two learning rates. In the dual learning rate model, the *Q*‐values were updated separately using $\alpha_{+}$ for positive prediction errors and $\alpha_{-}$ for negative prediction errors.

Model parameter estimation was performed using the Bayesian model Python package PyMC version 5.17.0 (Abril‐Pla et al. 2023). All models were run with four chains with 2000 tune (to optimize the MCMC sampler) and 4000 posterior samples each. Convergence between the Markov Chain Monte Carlo chains was assessed using the Gelman–Rubin statistic (Gelman and Rubin 1992), which was less than 1.1 for all estimates, indicating successful convergence.

To model the parameters α (or $\alpha_{+}$, $\alpha_{-}$), and β within the Bayesian framework, we employed a hierarchical, noncentered parameterization approach. Parameters were sampled with priors in the logit space (annotated by “~”) and transformed from the logit space to the original parameter space using a sigmoid function scaled by the maximum permissible value of the parameter, $\theta_{\max}$. This hierarchical model produced subject‐specific parameter values for each DBS condition by combining group‐ and subject‐level effects:

(11.1) $$\theta_{i,j}=\theta_{\max}\;f\left(\tilde{\mu }+z_{i}\;\tilde{\sigma }+j\left({\tilde{\mu }}^{\mathrm{DBS}}+z_{i}^{\mathrm{DBS}}\;{\tilde{\sigma }}^{DBS}\right)\right)$$

(11.2) $$f\left(x\right)=\frac{1}{1+e^{-x}}$$

where i indexes subjects and j indexes DBS (ON: j=1 or OFF: j=0).

The priors for group‐level mean $\tilde{\mu }$, standard deviation $\tilde{\sigma }$, DBS‐induced group‐level change ${\tilde{\mu }}^{\mathrm{DBS}}$, and standard deviation ${\tilde{\sigma }}^{\mathrm{DBS}}$ were modeled in the transformed logit space as normal and exponential distributions:

(12.1) $$\tilde{\mu }\sim N\left({\tilde{\mu }}_{\text{prior}},{\tilde{\sigma }}_{\text{prior}}^{\mu }\right)$$

(12.2) $${\tilde{\mu }}^{\mathrm{DBS}}\sim N\left(0,{\tilde{\sigma }}_{\text{prior}}^{\mu ,\mathrm{DBS}}\right)$$

(12.3) $$\tilde{\sigma }\sim \text{Exponential}\left(\frac{1}{{\tilde{\sigma }}_{\text{prior}}^{z}}\right)$$

(12.4) $${\tilde{\sigma }}^{\mathrm{DBS}}\sim \text{Exponent}i\mathrm{al}\left(\frac{1}{{\tilde{\sigma }}_{\text{prior}}^{z,\mathrm{DBS}}}\right)$$

where the parameters ${\tilde{\mu }}_{\text{prior}}$, ${\tilde{\sigma }}_{\text{prior}}^{\mu }$, ${\tilde{\sigma }}_{\text{prior}}^{\mu ,\mathrm{DBS}}$, ${\tilde{\sigma }}_{\text{prior}}^{z}$, and ${\tilde{\sigma }}_{\text{prior}}^{z,\mathrm{DBS}}$ of the prior distributions in the logit space were obtained by transforming the prior values in the original parameter space shown in Table 5 to the logit space.

**TABLE 5:** Priors of the TD parameters in original parameter space.

Parameter	$\mu_{\text{prior}}$	$\sigma_{\text{prior}}^{\mu }$	$\sigma_{\text{prior}}^{z}$	$\sigma_{\text{prior}}^{\mu ,\mathrm{DBS}}$	$\sigma_{\text{prior}}^{z,\mathrm{DBS}}$	$\theta_{\max}$
$\alpha_{+}$/$\alpha_{-}$	0.37	0.1	0.1	0.1	0.1	1.0
β	6.60	1.0	2.0	3.0	1.0	20.0

Subject‐level variations $z_{i}$ and $z_{i}^{\mathrm{DBS}}$ were drawn from a standard normal distribution:

(13.1) $$z_{i}\sim N\left(0,1\right)$$

(13.2) $$z_{i}^{\mathrm{DBS}}\sim N\left(0,1\right)$$

### 2.7 Statistics

For statistics, the Python package pingouin version 0.5.4 (Vallat 2018) was used. For *t*‐tests for unpaired samples (including post hoc *t*‐tests), data were checked for normal distribution using a Shapiro–Wilk test and for variance homogeneity using a Levene test. If requirements were not met, Welch's *t*‐tests were applied, with the uncorrected degrees of freedom indicated in the text. Linear regressions (mixed‐effects models) were applied using the Python package statsmodels version 0.15.0. For post hoc *t*‐tests and linear regression models, the Bonferroni correction for multiple tests was applied. Unless otherwise noted, a significance level of *α* = 0.05 was used.

## 3 Results

### 3.1 Habitual Biases Explaining Patient Data

**FIGURE 2:**
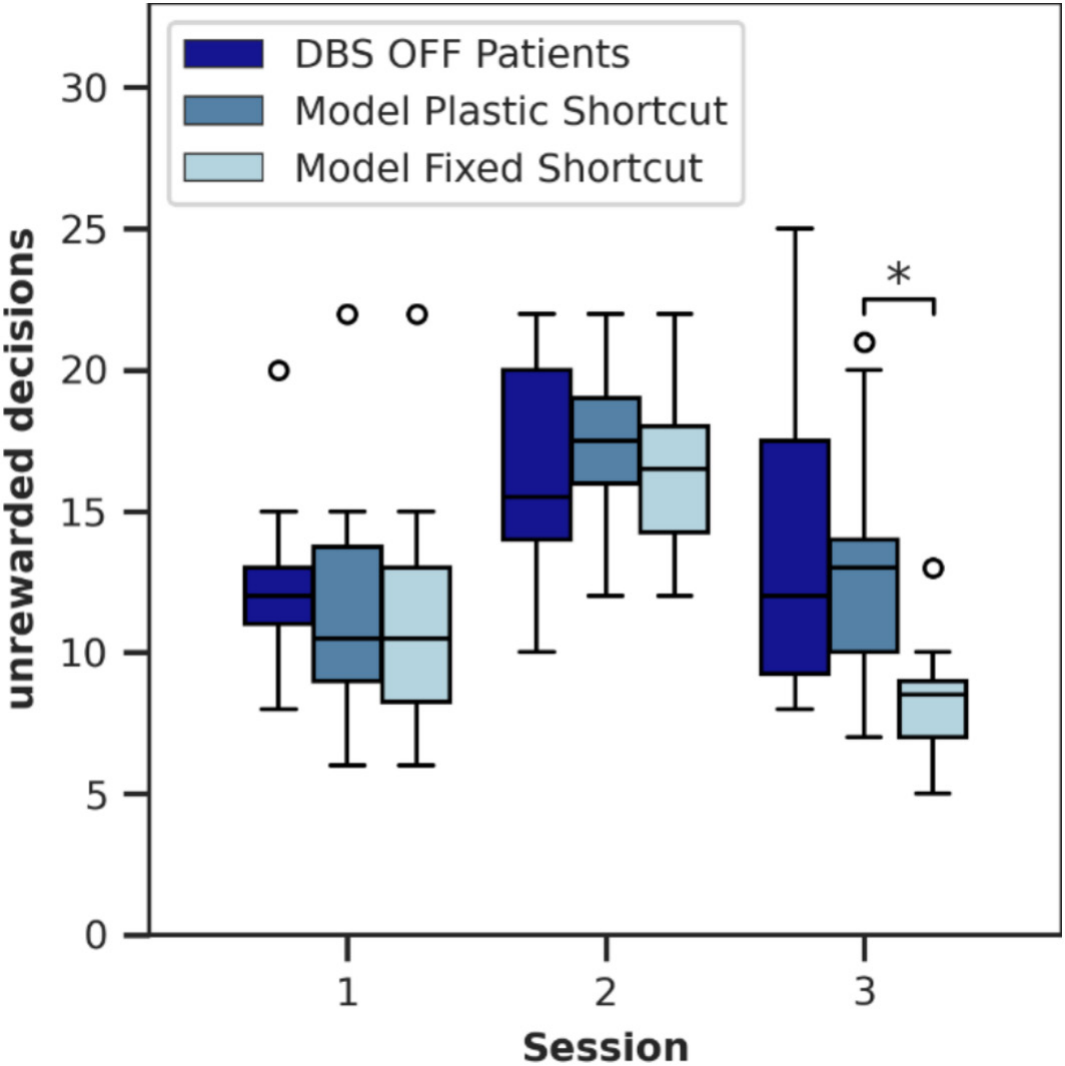
Number of unrewarded decisions in the three sessions of the task for the 14 patients from de A Marcelino et al. (2023) in the DBS OFF condition and the model with plastic and fixed cortico‐thalamic shortcut. Plasticity in the cortico‐thalamic shortcut promotes habit behavior, causing more unrewarded decisions, especially in the third session. Significant differences are annotated. For a more detailed time course of the unrewarded decisions, see Figure S1. The data of 14 patients/simulations are displayed as boxplots: horizontal line—the median, box—the interquartile range (IQR) from the 25th percentile to the 75th percentile, whiskers—extending up to 1.5 times the IQR, circles—outliers outside 1.5 times the IQR.

First, we adjusted specific parameters of our basal ganglia model (see Section S2) to replicate the behavioral data of patients in the DBS OFF condition from the study by de A Marcelino et al. (2023). In their study, patients performed a reversal learning task consisting of 120 trials. The reward contingencies for the two possible choices changed after 60 trials, with the task divided into three sessions of 40 trials each. Due to the reward reversal in the middle of Session 2, the number of unrewarded decisions increased during Session 2 and subsequently decreased in Session 3, though it remained higher than in Session 1. This pattern is also reflected in our model with a plastic cortico‐thalamic shortcut (see Figure 2). Figure S1 provides a more detailed time course of the unrewarded decisions, highlighting the increase precisely after the reward reversal.

By deactivating the plasticity of the cortico‐thalamic shortcut while keeping other conditions identical across 14 simulations, we observed a reduction in the number of unrewarded decisions exclusively during the third session compared with simulations with a plastic shortcut [two‐sided Welch's *t*‐test, *t*(26) = −3.60, *p* = 0.002]. This demonstrates that a plastic shortcut promotes habitual behavior, that is, biases the model toward choosing the previously most frequently selected decision. How the plastic shortcut learns to favor the initially frequently rewarded decision is shown in more detail in Section 3.5 and Figure S8.

This finding could also clarify the counterintuitive observation that patients made more unrewarded decisions in the third session than in the first session, despite the first session involving the uncertainty of initial learning of the reward contingencies. By the start of the third session, 20 trials had already been completed since the reversal, allowing the reward contingencies prevalent in Session 3 to be learned. Furthermore, if DBS increases the influence of the shortcut on decision‐making, it should further bias decisions toward habitual behavior. Consequently, we examined the effect of DBS on model behavior.

### 3.2 DBS Promoting Habitual Biases

In our study, we tested five DBS variants: *suppression*, *efferent*, *afferent*, *passing fibers*, and *combined*. For four of these variants (all except *afferent*), we could find parameters that replicated the DBS ON behavioral data of the patients of de A Marcelino et al. (2023) (see Section 3.8). Consequently, the *afferent* DBS variant was excluded from further analyses (Figure S7 also shows that it disrupts learning in the model).

**FIGURE 3:**
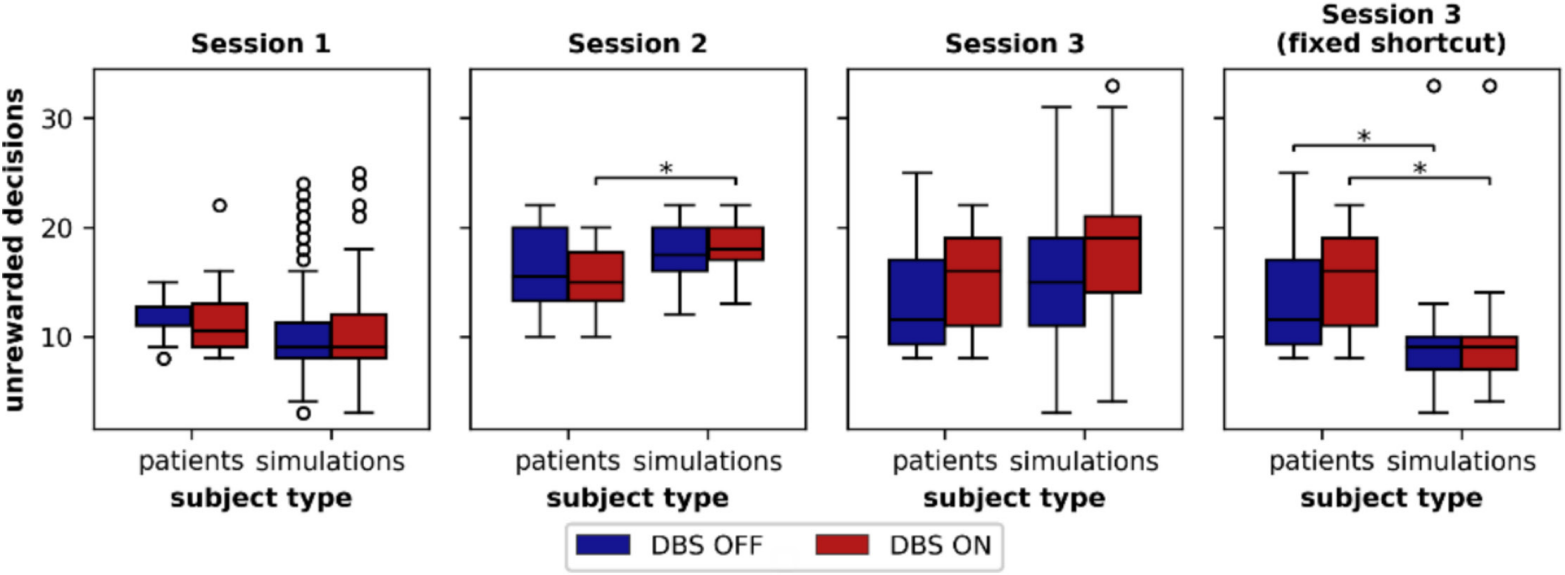
Comparison of unrewarded decisions between patients from de A Marcelino et al. (2023) and simulations under the combined DBS variant. The right panel displays the model with the fixed shortcut. Significant differences between patient data and simulations, as identified by post hoc *t*‐tests, are annotated. While the model with the plastic shortcut aligns well with the patient data, the model with the fixed shortcut shows substantial deviations, particularly in Session 3. In Figure S2, the data of all DBS variants of the model with a plastic shortcut are displayed, and in Figure S3, the data of the model with a fixed shortcut are displayed. The data of 14 patients/100 simulations are displayed as boxplots: horizontal line—the median, box—the interquartile range (IQR) from the 25th percentile to the 75th percentile, whiskers—extending up to 1.5 times the IQR, circles—outliers outside 1.5 times the IQR.

Figure 3 compares the model's unrewarded decisions under the *combined* DBS variant with the patients' unrewarded decisions. Due to the high similarity in unrewarded decisions across the DBS variants, only the *combined* variant is presented here. A comprehensive overview of all DBS variants in comparison with the patient data is available in Figure S2. Figure 3 shows that, also in the DBS ON condition, unrewarded decisions increase during Session 2 and remain higher in Session 3 compared with Session 1. Notably, Session 3 exhibits a potential increase in unrewarded decisions, which is further analyzed below.

To compare the model's unrewarded decisions data with the patients' data, we conducted a two‐way mixed ANOVA with the between factor subject type (patients and simulations) and the within factor DBS state (ON and OFF) for each session of the task. The results were consistent across all DBS variants. **Session 1**: No main effects for the subject type or DBS state and no interaction. **Session 2**: A main effect of subject type [*combined*: *F*(1, 112) = 11.513, *p* = 0.001; *efferent*: *F*(1, 112) = 10.172, *p* = 0.002; *passing fibers: F*(1, 112) = 10.282, *p* = 0.002; *suppression*: *F*(1, 112) = 10.977, *p* = 0.001] and an interaction [*combined*: *F*(1, 112) = 7.455, *p* = 0.007; *efferent*: *F*(1, 112) = 10.219, *p* = 0.002; *passing fibers*: *F*(1, 112) = 10.278, *p* = 0.002; *suppression*: *F*(1, 112) = 7.930, *p* = 0.006]. Post hoc *t*‐tests revealed that the model made significantly more unrewarded decisions than patients in the DBS ON condition [*combined*: *t*(112) = 3.413, *p* = 0.008; *efferent*: *t*(112) = 3.418, *p* = 0.007; *passing fibers*: *t*(112) = 3.391, *p* = 0.008; *suppression*: *t*(112) = 3.371, *p* = 0.008], with no significant difference in the DBS OFF condition. **Session 3**: A main effect of DBS state [*combined*: *F*(1, 111) = 23.211, *p* < 0.001; *efferent*: *F*(1, 111) = 29.167, *p* < 0.001; *passing fibers*: *F*(1, 111) = 5.912, *p* = 0.017; *suppression*: *F*(1, 111) = 26.109, *p* < 0.001].

In summary, the model replicates the patient data reasonably well, though Session 2 is less accurately represented due to the higher number of unrewarded decisions. However, the DBS effect in Session 3 aligned with the patient data (no interaction), where Figure 3 shows that the number of unrewarded decisions increases slightly due to DBS. This supports our hypothesis that the plastic cortico‐thalamic shortcut, which drives increased unrewarded decisions in Session 3 (see Section 3.1), gains influence under DBS.

To further assess the role of the plasticity of the shortcut, we simulated the DBS ON conditions using the model with the fixed shortcut. Results for Sessions 1 and 2 were comparable to those with the model with the plastic shortcut (see Figure S3), but Session 3 showed significant differences (see Figure 3 right). The two‐way mixed ANOVA for the model with the fixed shortcut revealed a main effect for subject type [*combined*: *F*(1, 111) = 30.679, *p* < 0.001; *efferent*: *F*(1, 111) = 30.702, *p* < 0.001; *passing fibers*: *F*(1, 111) = 31.618, *p* < 0.001; *suppression*: *F*(1, 111) = 31.491, *p* < 0.001] and DBS state [*combined*: *F*(1, 111) = 15.096, *p* < 0.001; *efferent*: *F*(1, 111) = 5.011, *p* = 0.027; *passing fibers*: *F*(1, 111) = 10.016, *p* = 0.002; *suppression*: *F*(1, 111) = 12.162, *p* < 0.001], along with an interaction [*combined*: *F*(1, 111) = 10.775, *p* = 0.001; *efferent*: *F*(1, 111) = 9.552, *p* = 0.003; *passing fibers*: *F*(1, 111) = 18.059, *p* < 0.001; *suppression*: *F*(1, 111) = 14.461, *p* < 0.001] for Session 3. Post hoc *t*‐tests revealed that the model with the fixed shortcut made significantly fewer unrewarded decisions in both DBS ON [*combined*: *t*(111) = −4.465, *p* = 0.001; *efferent*: *t*(111) = −4.589, *p* < 0.001; *passing fibers*: *t*(111) = −4.602, *p* < 0.001; *suppression*: *t*(111) = −4.547, *p* = 0.001] and DBS OFF [*combined*: *t*(111) = −3.047, *p* = 0.019; *efferent*: *t*(111) = −3.047, *p* = 0.019; *passing fibers*: *t*(111) = −3.047, *p* = 0.019; *suppression*: *t*(111) = −3.047, *p* = 0.019] conditions. In summary, only the model with the plastic shortcut accurately captured the data on unrewarded decisions and the DBS effect in Session 3.

**FIGURE 4:**
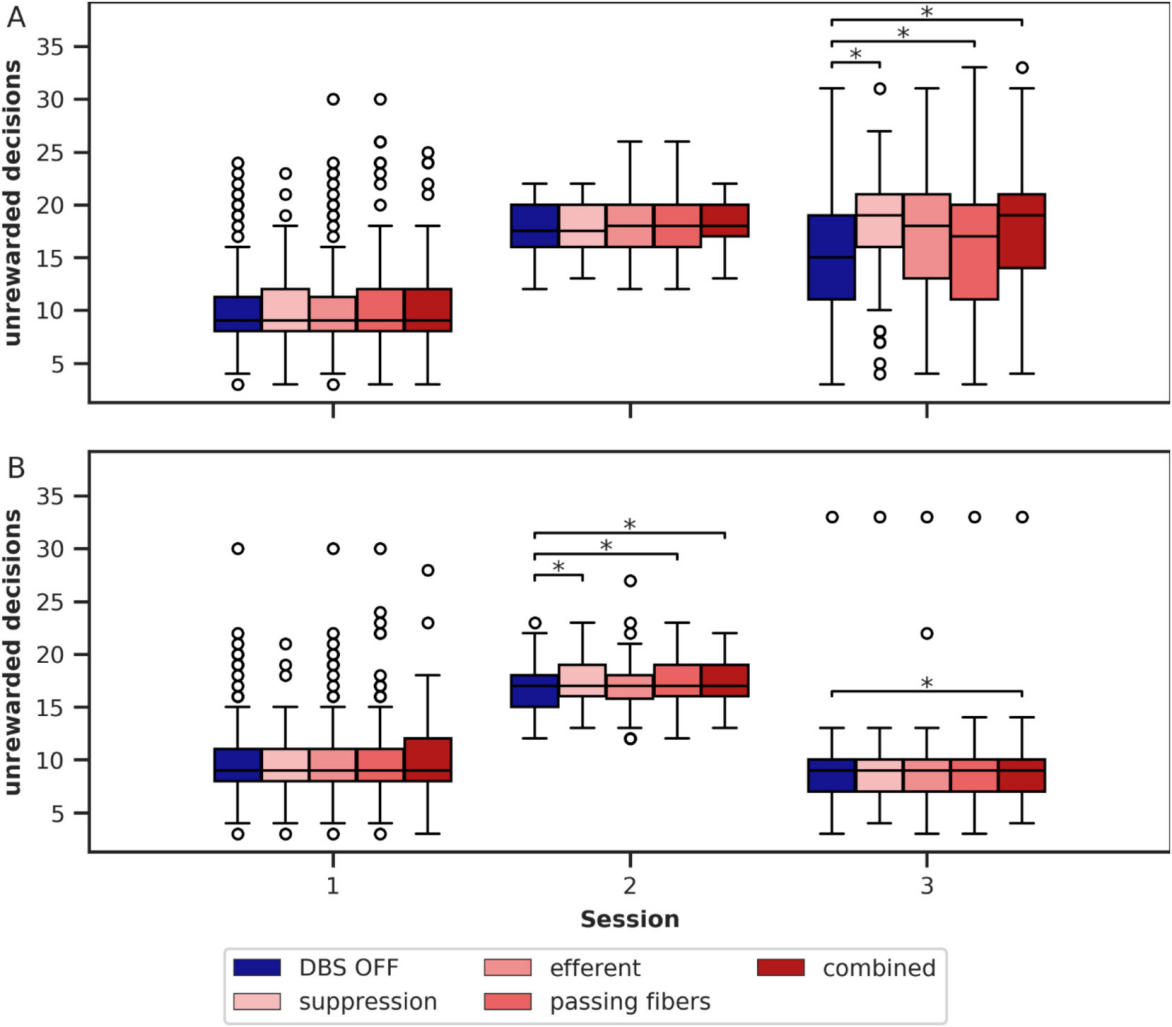
Comparison of unrewarded decisions between the DBS variants. Significant effects of DBS (compared with DBS OFF) are annotated. (A) Model with the plastic shortcut, (B) model with the fixed shortcut. In the model with the plastic shortcut, all DBS variants except *passing fibers* increase significantly the unrewarded decisions in Session 3. In the model with the fixed shortcut, the unrewarded decisions in Session 3 are, in general, much lower, and DBS effects are much weaker and only present for the DBS variant *combined*. The data of 100 simulations are displayed as boxplots: horizontal line—the median, box—the interquartile range (IQR) from the 25th percentile to the 75th percentile, whiskers—extending up to 1.5 times the IQR, circles—outliers outside 1.5 times the IQR.

The analyses so far compared the model data with patient data, revealing no differences between the DBS variants. Next, we examine the specific effects of DBS on unrewarded decisions across the different DBS variants in greater detail and compare them with one another. Figure 4 illustrates the model's unrewarded decisions across the DBS variants.

To analyze the effects of the DBS variants on unrewarded decisions, we fitted a linear regression model for each session, using unrewarded decisions as the dependent variable, the categorical fixed effect “DBS variant” (*DBS OFF*, *suppression*, *efferent*, *passing fibers*, and *combined*), and random intercepts for the simulations. *DBS OFF*, which is identical for all variants, served as the baseline category. The estimated coefficients and their statistical values are presented in Table 6 for the model with the plastic shortcut and in Table 7 for the model with the fixed shortcut.

**TABLE 6:** Linear regression model results for the model with the plastic shortcut.

Session	DBS variant	Coefficient	*z* values	*p*	*p* corrected	[0.025]	[0.975]
1	*Combined*	−0.06	−0.271	0.787	1.000	−0.494	0.374
	*Efferent*	0.17	0.767	0.443	1.000	−0.264	0.604
	*Passing fibers*	0.26	1.173	0.241	0.963	−0.174	0.694
	*Suppression*	−0.34	−1.534	0.125	0.500	−0.774	0.094
2	*Combined*	0.16	1.274	0.203	0.810	−0.086	0.406
	*Efferent*	0.19	1.513	0.130	0.521	−0.056	0.436
	*Passing fibers*	0.16	1.274	0.203	0.810	−0.086	0.406
	*Suppression*	0.13	1.035	0.301	1.000	−0.116	0.376
3	*Combined*	2.72	5.119	< 0.001	< 0.001	1.679	3.761
	*Efferent*	2.10	3.952	< 0.001	< 0.001	1.059	3.141
	*Passing fibers*	0.88	1.656	0.098	0.391	−0.161	1.921
	*Suppression*	2.86	5.382	< 0.001	< 0.001	1.819	3.901

**TABLE 7:** Linear regression model results for the model with the fixed shortcut.

Session	DBS variant	Coefficient	*z* values	*p*	*p* corrected	[0.025]	[0.975]
1	*Combined*	−0.17	−0.738	0.46	1.000	−0.621	0.281
	*Efferent*	0.00	0.000	1.000	1.000	−0.451	0.451
	*Passing fibers*	0.06	0.261	0.794	1.000	−0.391	0.511
	*Suppression*	−0.46	−1.997	0.046	0.183	−0.911	−0.009
2	*Combined*	0.60	5.026	< 0.001	< 0.001	0.366	0.834
	*Efferent*	0.12	1.005	0.315	1.000	−0.114	0.354
	*Passing fibers*	0.40	3.351	0.001	0.003	0.166	0.634
	*Suppression*	0.46	3.854	< 0.001	< 0.001	0.226	0.694
3	*Combined*	0.40	3.142	0.002	0.007	0.150	0.650
	*Efferent*	0.20	1.571	0.116	0.465	−0.050	0.450
	*Passing fibers*	0.21	1.649	0.099	0.396	−0.040	0.460
	*Suppression*	0.29	2.278	0.023	0.091	0.040	0.540

In the model with the plastic shortcut, all DBS variants except *passing fibers* significantly increased the number of unrewarded decisions in Session 3. For Sessions 1 and 2, no significant effects are present. In the model with the fixed shortcut, the increases in Session 3 were absent, except for the *combined* DBS variant, which had a significant but substantially smaller coefficient than in the model with the plastic shortcut. Additionally, in the model with the fixed shortcut, small but significant increases in unrewarded decisions were observed in Session 2.

In summary, the DBS variants *suppression*, *efferent*, and *combined* align with our hypothesis that DBS amplifies the influence of the plastic shortcut, as reflected by the increased number of unrewarded decisions in Session 3 (as in Section 3.1). This amplifies the habitual biases in decision‐making, that is, favors the previously frequently rewarded decision, and consequently increases the number of unrewarded decisions. In contrast, the fixed shortcut model does not learn any habitual biases. Although it is slightly impaired in its ability to relearn through DBS, it lacks the mechanism that further promotes habitual behavior.

### 3.3 DBS Increasing P(Explore)

de A Marcelino et al. (2023) proposed that DBS in the GPi promotes exploratory behavior. They concluded this by estimating the parameters of a temporal difference (TD) learning model, which evaluates the values of the two available options, combined with a drift diffusion model to map the data of the individual patients. They defined exploratory behavior as nongreedy choices, specifically, selecting the option with the lower value according to the fitted TD algorithm. Based on this approach, they calculated the probability of exploratory choices, P(Explore), as the proportion of the low‐value choices relative to all choices during a session.

We adopt a similar approach to estimate P(Explore), using a TD learning model combined with softmax action selection to replicate decisions (but not response times). The model updates option values (*Q*‐values) iteratively using the delta learning rule (see Equation 10.1). As in de A Marcelino et al. (2023), we tested two variants: a single learning rate model and a dual‐rate model with separate learning rates for positive and negative prediction errors.

**FIGURE 5:**
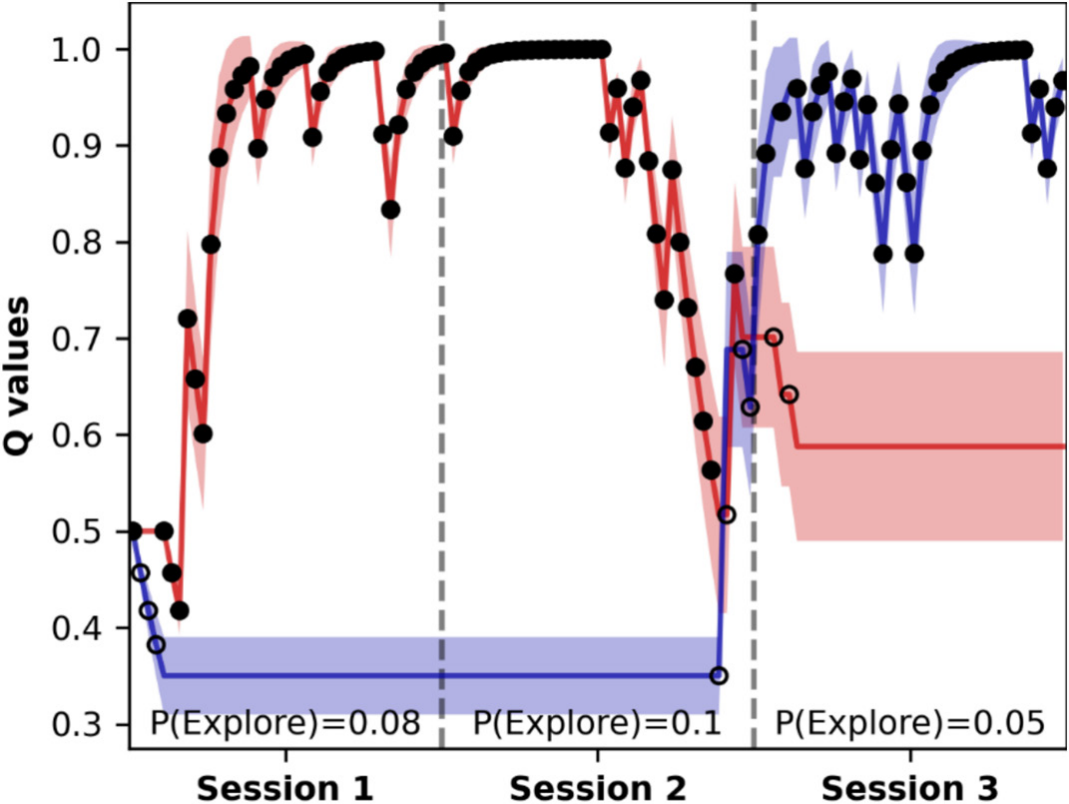
Example of expected *Q*‐values and P(Explore) estimation. The expected *Q*‐values of both options (option 1 in red and option 2 in blue) are obtained by averaging over the 4000 posterior samples. The transparent area represents the standard deviation. Filled circles show high‐value choices and empty circles show low‐value choices.

We estimated the parameters of the TD model using a hierarchical Bayesian model, separately fitting patient and simulation data. The simulations included only the DBS variants *suppression*, *efferent*, and *combined*, which showed significantly increased unrewarded decisions in Session 3 (see Section 3.2). Consistent with de A Marcelino et al. (2023), the dual‐rate model provided a better fit than the single‐rate model (evaluated via leave‐one‐out cross‐validation; see Figure S4). Using the inferred parameter distributions, we calculated the expected *Q*‐values for the two available options across the task and computed P(Explore) for each subject (patient/simulation) by session and DBS state (e.g., see Figure 5).

**FIGURE 6:**
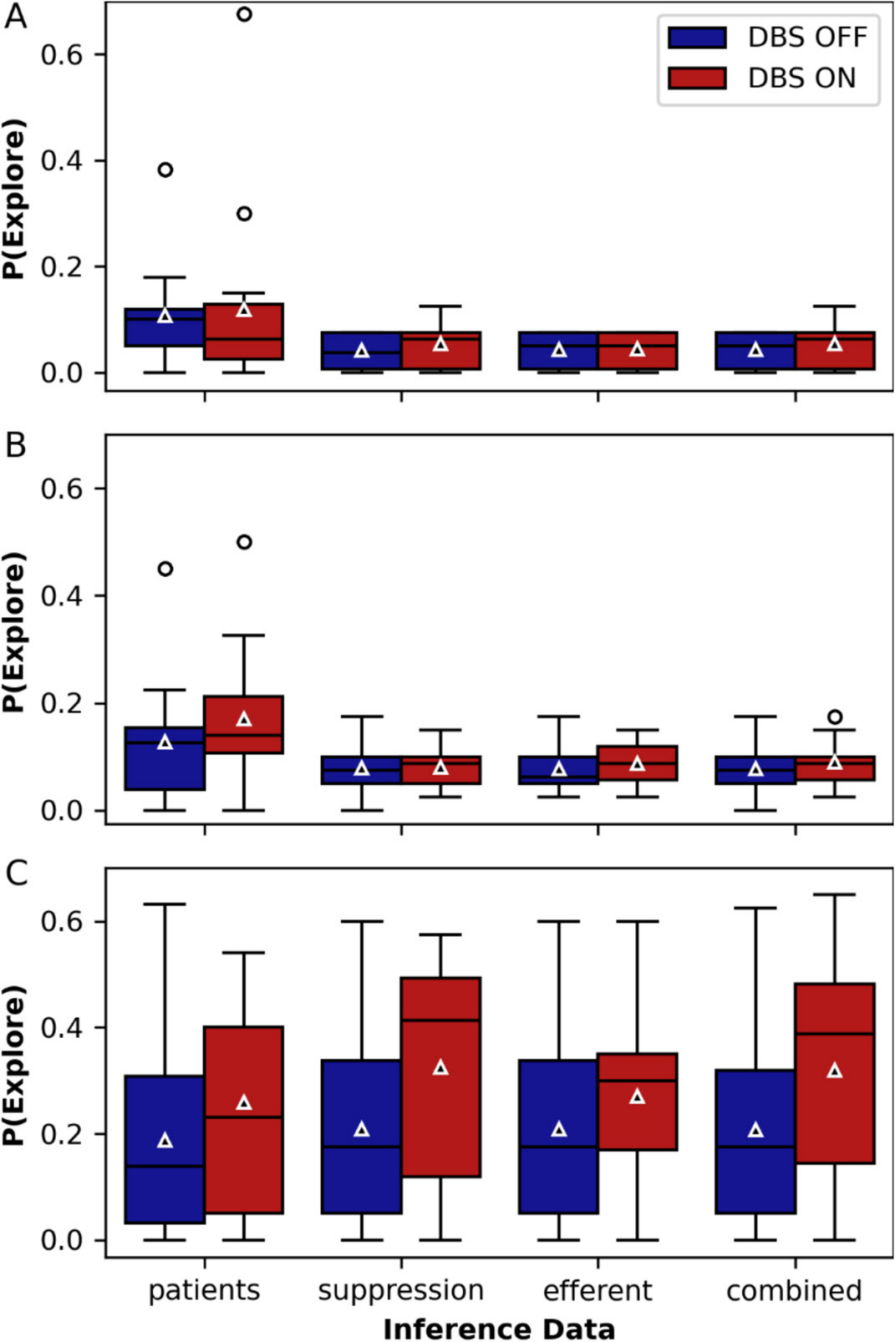
P(Explore) inferred from patients and simulations under DBS variants *suppression*, *efferent*, and *combined*. (A) Session 1, (B) Session 2, (C) Session 3. In Sessions 1 and 2, P(Explore) inferred from the simulations is lower than P(Explore) inferred from the patients. In Session 3, all simulated DBS variants align with the patient data. In Session 3, DBS consistently increases P(Explore). The data of 100 simulations are displayed as boxplots: horizontal line—the median, triangle—the mean, box—the interquartile range (IQR) from the 25th percentile to the 75th percentile, whiskers—extending up to 1.5 times the IQR, circles—outliers outside 1.5 times the IQR.

To assess the effect of DBS on P(Explore) and compare patients with simulations, we conducted a mixed two‐way ANOVA for each session with the between factor Inference data (patients, suppression, efferent, and combined), reflecting the data used for parameter inference and P(Explore) estimation, and the within factor DBS state (ON and OFF). In Session 1, the ANOVA revealed a significant main effect for Inference data [*F*(3, 52) = 2.979, *p* = 0.040], where Figure 6 shows that P(Explore) inferred from the simulation data was lower than that inferred from patient data. However, no significant main effect of DBS state or interaction between the factors was observed. In Session 2, the ANOVA revealed significant main effects for both Inference data and DBS state [inference data: *F*(3, 52) = 3.472, *p* = 0.022; DBS state: *F*(1, 52) = 5.257, *p* = 0.026], with no significant interaction between the factors. As shown in Figure 6, P(Explore) remained lower for simulation data compared with patient data, and there was an overall increase in P(Explore) due to DBS activation. In Session 3, the ANOVA revealed only a significant main effect of DBS state [*F*(1, 51) = 13.677, *p* < 0.001]. Figure 6 illustrates that DBS increased P(Explore) independently of whether the data was inferred from patients or simulations.

In summary, our model replicates the DBS‐induced increase in P(Explore), particularly in Session 3, although it shows a slightly lower P(Explore) than the patients in Sessions 1 and 2. The DBS effect on P(Explore) is independent of the DBS variant used, with all variants replicating patient data equally well.

The simultaneous increase in both habitual and exploratory behavior in the model presents a contradiction. This suggests that the method used to calculate P(Explore) in this task does not effectively differentiate between underlying exploratory and habitual behavior. Habitual behavior increases the likelihood of repeating previously rewarded decisions, while exploration leads to more random decision‐making. Both tendencies ultimately reduce the probability of consistently selecting the option with the highest value (according to the TD algorithm). As our model implements a habitual bias and leads to similar P(Explore) values as human subjects, the conclusion that human behavior reflects increased exploration is not conclusive.

### 3.4 Activity Changes Induced by DBS

**FIGURE 7:**
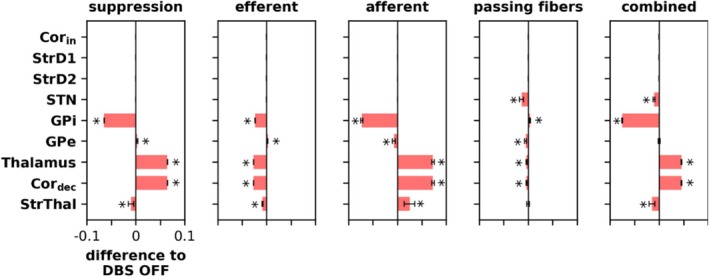
Average absolute rate changes in the populations of the untrained model caused by the different DBS variants. The corresponding values are shown in Table S8. The average rates in the DBS OFF condition are shown in the Figure S5, and an overview of the rates in all conditions is shown in Table S9. Significant activity changes were obtained using a linear regression model (with the categorical fixed effect “DBS variant” (*DBS OFF*, *suppression*, *efferent*, *afferent*, *passing fibers*, and *combined*), *DBS OFF* as baseline, random intercepts for the simulations) and are annotated with an asterisk (*). Bar lengths show the average over 100 simulations with error bars showing the SD. Each simulation consisted of a single trial, thus simulating the untrained model, where the rates were calculated from the time window 2500–3000 ms.

In addition to analyzing the model's behavior in the reversal learning task, we examined the changes in individual population activity induced by DBS. To do this, we compared the average firing rates of all populations of the untrained model (initial state) between DBS ON and OFF for all DBS variants (Figure 7).

Under normal conditions, the model supports a particular choice by letting one GPi neuron have significantly reduced activity compared with the other. The *suppression* DBS variant inhibits both GPi neurons, resulting in an average decrease in GPi activity, which subsequently increases activity in the thalamus and Cor_dec_. Unexpectedly, the activity of the StrThal population decreases. This occurs because the already less active GPi neuron increases its activity slightly (instead of decreasing it) due to less competition from the other, now suppressed, GPi neuron, which outweighs the additional suppression from DBS. Consequently, the activity of the thalamus and Cor_dec_ neurons linked to the already less active GPi neuron decreases, as does the activity of the StrThal neuron, which is the only active neuron in StrThal, unlike in the thalamus and Cor_dec_. The slight decrease in StrThal activity leads to a slight increase in GPe activity.

The *efferent* DBS variant increases the overall output of the GPi, resulting in stronger inhibition of the thalamus and reduced activity in the Cor_dec_ and StrThal. The slight decrease in StrThal activity leads to a slight increase in GPe activity. The activity of GPi decreases due to enhanced competition caused by the increased output of all GPi neurons.

The *afferent* DBS variant enhances all GPi inputs, leading to greater inhibition of the GPi on average. This disinhibits the thalamus, resulting in higher activity in the Cor_dec_ and StrThal and a slight increase in GPe inhibition by StrThal.

The *passing fibers* DBS variant slightly reduces STN activity, while the *combined* DBS variant combines the effects of *suppression*, *efferent*, and *passing fibers*.

In summary, different DBS variants distinctly affect individual population activities. Notably, *suppression* and *efferent* variants have vastly different effects on population activities but produce similar behavioral outcomes (see Sections 3.2 and 3.3).

**FIGURE 8:**
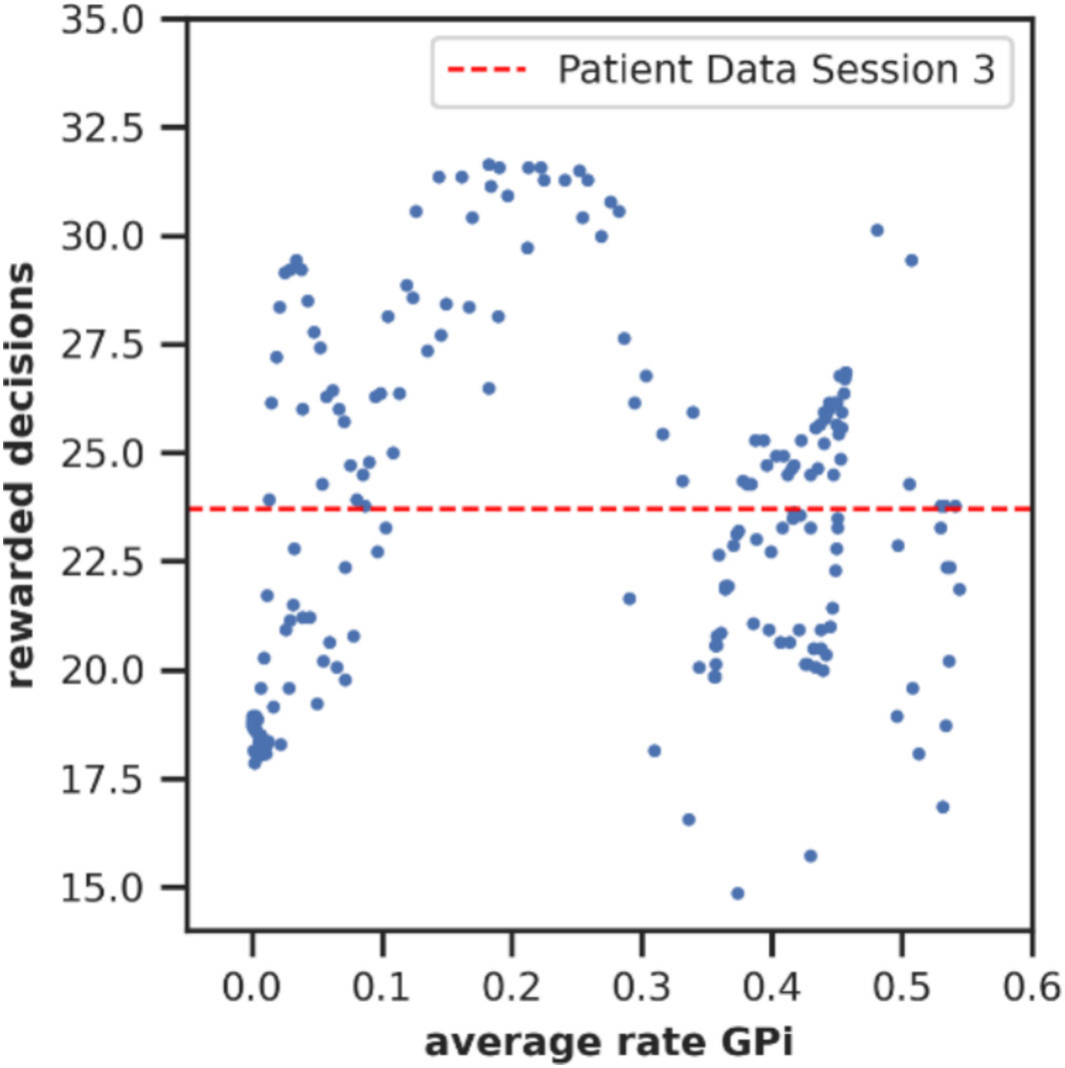
Relationship between the average GPi firing rate and the number of rewarded decisions in Session 3. Each point represents data corresponding to a unique DBS parameter set derived from the parameter search for the DBS variants (Section 3.8). For each parameter set, 14 simulations were performed. As in Figure 7, for each simulation, GPi firing rates were obtained from the untrained model (i.e., the first trial of the simulation) from the time window of 2500–3000 ms. The number of rewarded decisions was obtained from Session 3 for each simulation. The points in the plot display the averages of these 14 firing rates and rewarded decision counts. The red dashed line shows the average number of rewarded decisions for the human patients (*n* = 14) in the third session of the task from de A Marcelino et al. (2023). The effects of the individual DBS variants on GPi firing rate and model behavior are shown in Figure S6.

A common feature of all DBS variants is GPi inhibition (apparent in *passing fibers* only at higher parameter values than shown in Figure 7; see Figure S6). We investigated the relationship between GPi activity and the number of rewarded decisions in Session 3 using data from the parameter search for the DBS variants (see Section 3.8). Various combinations of GPi rate and model performance were observed across different DBS variants (Figure 8). However, no simple linear relationship exists. Similar GPi rates correspond to a wide range of performance values. Thus, other DBS effects on the model cannot be ignored, and GPi inhibition cannot be considered the sole mechanism of DBS action, at least based on our model.

### 3.5 Pathway‐Specific Dynamics of Plastic Connections

The model's behavior depends critically on the development of the plastic connections associated with the direct, indirect, and hyperdirect pathways and the cortico‐thalamic shortcut. Each pathway modulates the influence of the Cor_in_ neurons on the two output Cor_dec_ neurons. To quantify these effects, the connectivity matrices of successive plastic connections were combined through matrix multiplication (only for the basal ganglia pathways), providing a direct estimate of the Cor_in_ neurons' influence on the output neurons. Given that the Cor_in_ neurons activate together during the task and consequently learn identical connectivity patterns, we averaged over the Cor_in_ neuron dimension. This yielded two values per trial for each pathway (including the shortcut), representing the influence of the Cor_in_ neurons on the two outputs.

**FIGURE 9:**
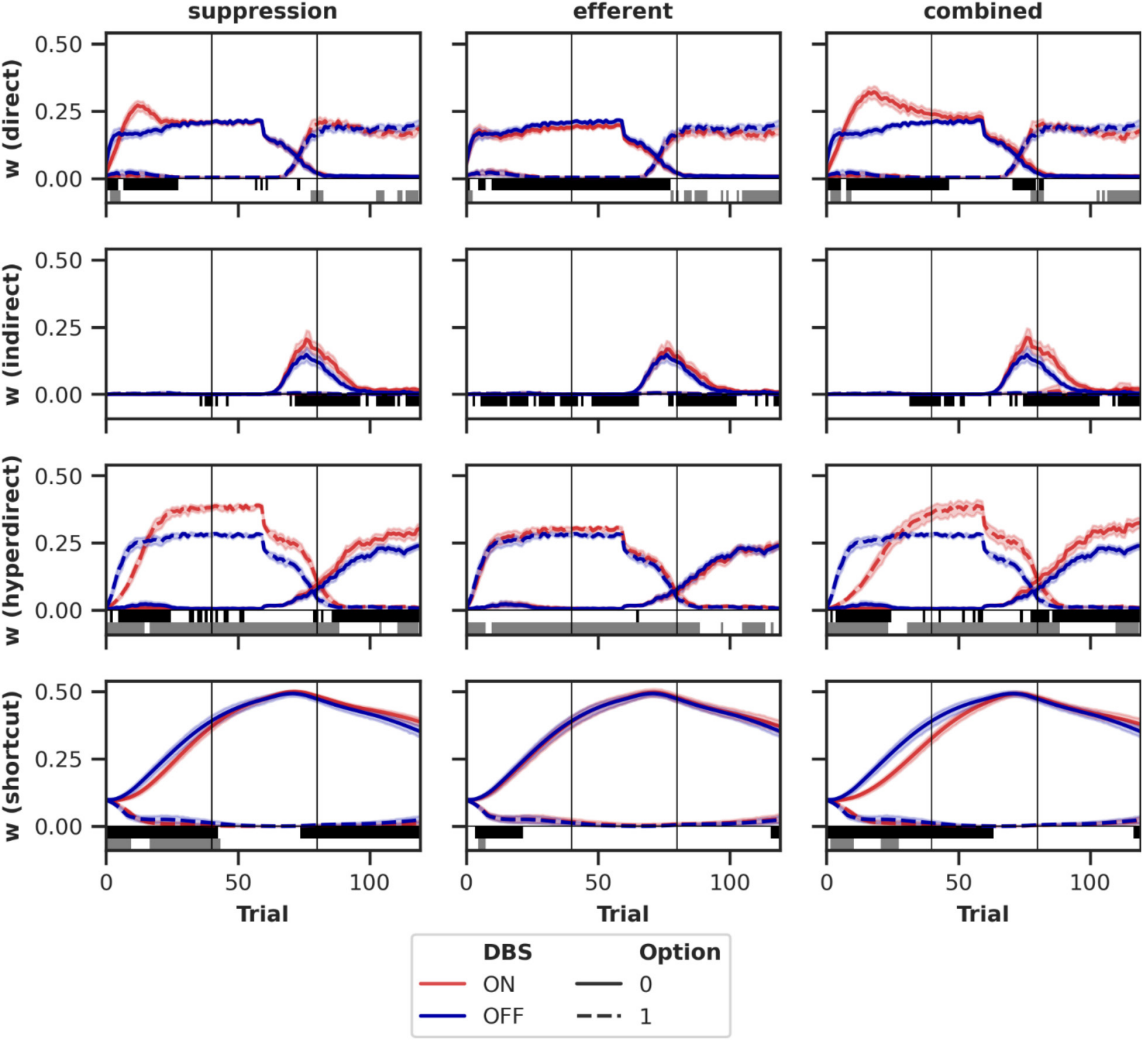
Average calculated weights from the Cor_in_ to the two output neurons in Cor_dec_. Each row displays a different plastic pathway and each column a different DBS variant. The weights for the DBS variants *afferent* and *passing fibers* are shown in Figure S7. The vertical lines indicate the session boundaries. The weights are averaged over 100 simulations (in each DBS condition); the transparent area displays the 95% confidence interval. Above the *x*‐axis, significant differences between DBS ON and OFF are indicated for output 0 in black and output 1 in gray. *p*‐values were corrected for multiple comparisons using the Benjamini–Hochberg procedure (Benjamini and Hochberg 1995).

Figure 9 illustrates the influence of the plastic pathways on the outputs across trials. We analyzed the progression for DBS OFF and the three DBS variants *suppression*, *efferent*, and *combined*, all leading to significantly more unrewarded decisions during Session 3 (see Section 3.2). A consistent pattern emerges across all DBS variants. The direct pathway initially learns to favor option 0 and, following reward reversal, shifts to favor option 1. The indirect pathway temporarily suppresses option 0 after the reward reversal. The hyperdirect pathway first suppresses alternatives to option 0 (i.e., suppressing option 1) and, after the reward reversal, suppresses alternatives to option 1 (i.e., suppressing option 0). The cortico‐thalamic shortcut gradually learns to favor option 0 and slowly unlearns this preference after the reversal. Option 0 refers to the decision frequently rewarded prior to the reward reversal, while option 1 refers to the decision frequently rewarded afterward.

Some DBS effects on the progression of connectivity align with the observation of increased unrewarded decisions in Session 3, for example, the slightly enhanced shortcut and weakened direct pathway at the end of Session 3, which should bias the model toward favoring the previously rewarded option (via the enhanced shortcut) and choosing less effectively the newly rewarded option (due to the weakened direct pathway). However, countervailing effects are also evident, such as the enhanced direct pathway shortly after the reward reversal and the enhanced indirect and hyperdirect pathways, which should suppress the previously rewarded option more strongly. The DBS variant *efferent* differs from the other two variants by exhibiting a weakened direct pathway throughout the whole Session 3, rather than a temporarily strengthened one, and by lacking a strengthened hyperdirect pathway. The effects of the *afferent* and *passing fibers* DBS variants are illustrated in Figure S7, which clearly demonstrates how the *afferent* variant significantly disrupts learning in the model.

In summary, a similar pattern emerges as with the activity changes. DBS significantly influences the progression of connectivity. However, the effects differ between DBS variants despite their similar behavioral outcomes and do not provide a clear explanation for the increased number of unrewarded decisions observed in Session 3.

### 3.6 DBS‐Induced Input Modulations in the Thalamus

Neither the activity changes nor the plasticity modulation induced by DBS alone provide a clear explanation for how the DBS variants *suppression*, *efferent*, and *combined* increase the influence of the shortcut and lead to the higher number of unrewarded decisions observed in Session 3 (Section 3.2). This indicates that the effects are likely due to a combination of activity changes and modulated plasticity. To explore this, we analyzed the input dynamics of the thalamic neurons in the model.

In the thalamus, inputs from the basal ganglia (via GPi) and the shortcut (via Cor_in_) converge. We quantified the difference between the two thalamic neurons for each of these inputs, as the balance of their activation is critical for decision‐making in the model. This difference provides a measure, hereafter referred to as *support*, indicating how strongly each input source (basal ganglia and shortcut) contributes to the selection of a specific option. For example, if the shortcut exclusively excites thalamic neuron 0, it provides positive support for selecting option 0 and negative support for selecting option 1. For basal ganglia support, the difference in thalamic inputs (from GPi) was multiplied by −1 to account for its inhibitory effect.

**FIGURE 10:**
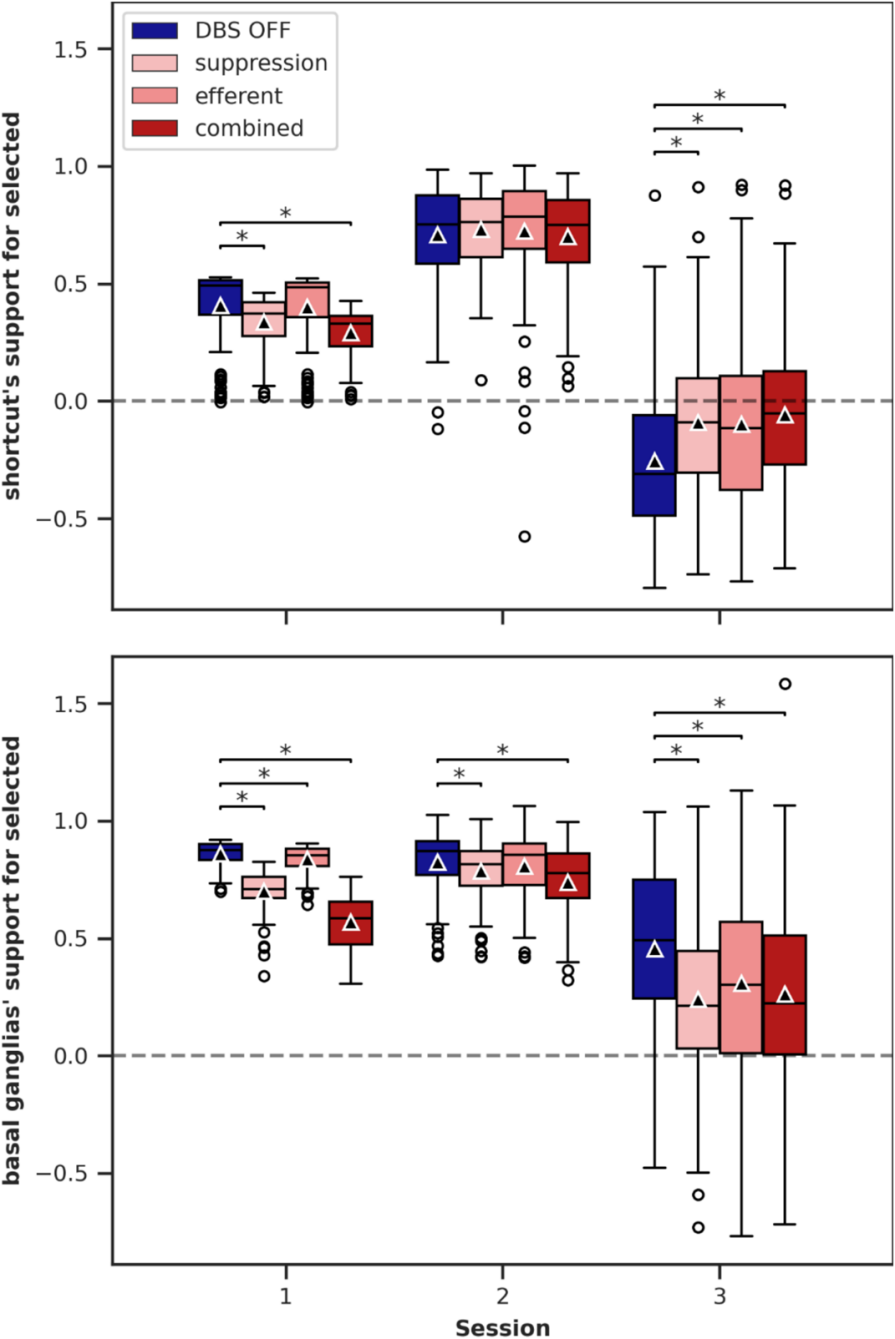
Support of the shortcut and basal ganglia in the thalamus for the selected option. Significant DBS effects (compared with DBS OFF) are annotated. Notably, in Session 3, the shortcut support increases significantly while the basal ganglia support decreases significantly. The same data for the model with the fixed shortcut is shown in Figure S9. The support exclusively for the initially frequently rewarded option is shown in Figure S8. The data of 100 simulations are displayed as boxplots: horizontal line—the median, triangle—the mean, box—the interquartile range (IQR) from the 25th percentile to the 75th percentile, whiskers—extending up to 1.5 times the IQR, circles—outliers outside 1.5 times the IQR.

To analyze the effects of the DBS variants on the support, we fitted a linear regression model for each session, using support as the dependent variable, the categorical fixed effect “DBS variant” (*DBS OFF*, *suppression*, *efferent*, and *combined*), and random intercepts for the simulations. *DBS OFF*, which is identical for all variants, served as the baseline category. The estimated coefficients and their statistical values are presented in Table 8 for the shortcut support and Table 9 for the basal ganglia support. Figure 10 visualizes the support values for both.

**TABLE 8:** Linear regression model results for shortcut support.

Session	DBS variant	Coefficient	*z* values	*p*	*p* corrected	[0.025]	[0.975]
1	*Combined*	−0.115	−13.318	< 0.001	< 0.001	−0.132	−0.098
	*Efferent*	−0.008	−0.895	0.371	1.000	−0.025	0.009
	*Suppression*	−0.070	−8.064	< 0.001	< 0.001	−0.086	−0.053
2	*Combined*	−0.007	−0.400	0.689	1.000	−0.04	0.026
	*Efferent*	0.016	0.932	0.352	1.000	−0.017	0.048
	*Suppression*	0.025	1.505	0.132	0.397	−0.008	0.058
3	*Combined*	0.199	6.044	< 0.001	< 0.001	0.134	0.263
	*Efferent*	0.157	4.777	< 0.001	< 0.001	0.093	0.221
	*Suppression*	0.166	5.051	< 0.001	< 0.001	0.102	0.231

**TABLE 9:** Linear regression model results for basal ganglia support.

Session	DBS variant	Coefficient	*z* values	*p*	*p* corrected	[0.025]	[0.975]
1	*Combined*	−0.290	−35.587	< 0.001	< 0.001	−0.306	−0.274
	*Efferent*	−0.023	−2.834	0.005	0.014	−0.039	−0.007
	*Suppression*	−0.159	−19.501	< 0.001	< 0.001	−0.175	−0.143
2	*Combined*	−0.086	−6.225	< 0.001	< 0.001	−0.113	−0.059
	*Efferent*	−0.018	−1.285	0.199	0.596	−0.045	0.009
	*Suppression*	−0.038	−2.752	0.006	0.018	−0.065	−0.011
3	*Combined*	−0.193	−5.479	< 0.001	< 0.001	−0.261	−0.124
	*Efferent*	−0.148	−4.214	< 0.001	< 0.001	−0.217	−0.079
	*Suppression*	−0.215	−6.114	< 0.001	< 0.001	−0.284	−0.146

In general, basal ganglia support is consistently high and positive, indicating its strong involvement in decision‐making. However, this support slightly decreases in Session 3. For the shortcut, support starts lower in Session 1, increases in Session 2 to levels comparable to the basal ganglia, and drops significantly in Session 3. This drop occurs because the shortcut continues to support the previously rewarded option, which is no longer frequently chosen as the basal ganglia quickly adapt to the reward reversal (see Figure S8 for support exclusively for the initially frequently rewarded option).

DBS has significant effects on support for the selected options. In Sessions 1 and 2, DBS primarily reduces basal ganglia support, with a smaller reduction observed for the shortcut. In Session 3, the effects are particularly notable: basal ganglia support is significantly reduced across all DBS variants, while shortcut support is significantly increased. For the fixed shortcut model, shortcut support is consistently zero by definition. In this model, basal ganglia support remains significantly higher compared with the plastic shortcut model, although DBS still induces slight reductions (see Figure S9).

In summary, before the reward reversal, the plastic shortcut and basal ganglia complement each other in guiding decision‐making. After the reversal, they compete: the shortcut continues to support the previously rewarded option, while the basal ganglia adapt quickly to the new reward contingencies and dominate decision‐making. DBS reduces basal ganglia influence in both the plastic and fixed shortcut models. However, in the plastic shortcut model, the reduction in basal ganglia influence is far more pronounced, allowing the competing shortcut to gain significant influence. This shift from basal ganglia to shortcut influence ultimately explains the increased tendency for habitual behavior observed in Session 3.

### 3.7 Disentangling Acute Activity and Progressive Plasticity Effects on Behavior

The previous sections demonstrate that the DBS variants *suppression*, *efferent*, and *combined* all increase the influence of the shortcut on decision‐making by modulating both neuronal activity and projection plasticity. This raises the question of how these two mechanisms interact to shape behavior.

To address this, we conducted 100 simulations for each DBS condition (*DBS OFF*, *suppression*, *efferent*, and *combined*) and saved the model states at the beginning of each trial in Session 3. These saved states allowed us to reload the model and continue the simulation from those trials under two experimental conditions: (1) with DBS ON and (2) with DBS OFF. This setup resulted in four groups of trials for each DBS variant, defined by two factors: (1) the *history* condition, representing the loaded model state (DBS ON or OFF), and (2) the *acute* condition, representing the current DBS state during the trial (DBS ON or OFF).

**FIGURE 11:**
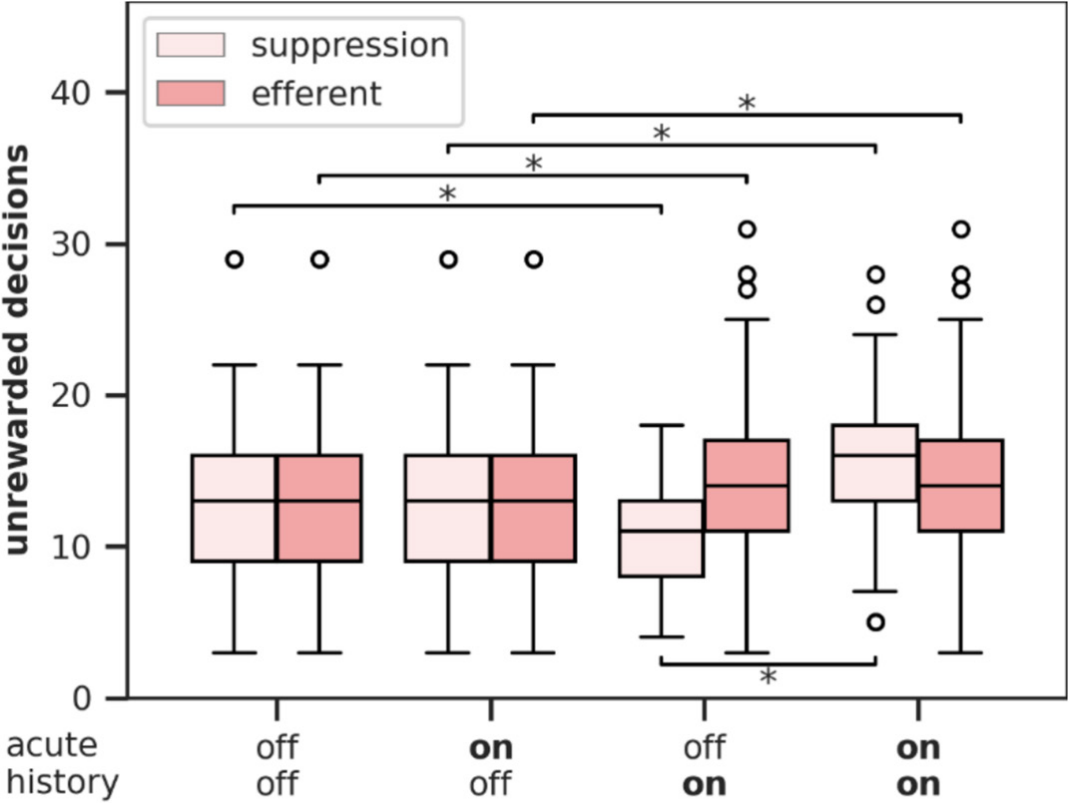
Number of unrewarded decisions in the third session of the task depending on whether DBS was applied during trials (acute) and whether DBS was applied in all trials before (history). Significant effects of acute and history, as identified by post hoc *t*‐tests, are annotated, for acute below the boxplots and for history above the boxplots. Exclusively applying DBS during trials (acute = on) without applying it in all trials before (history = off) does not lead to any behavioral changes (compared with both off). The DBS variant *combined* is shown in Figure S10. The data of 100 simulations are displayed as boxplots: horizontal line—the median, box—the interquartile range (IQR) from the 25th percentile to the 75th percentile, whiskers—extending up to 1.5 times the IQR, circles—outliers outside 1.5 times the IQR.

This design enabled us to disentangle the effects of DBS on unrewarded decisions by separating the influence of progressively modulated plasticity (*history*) from the influence of acutely modulated neuronal activity (*acute*). Figure 11 shows the unrewarded decisions across the acute and history factors for the *suppression* and *efferent* DBS variants. The *combined* variant is not shown because its results are nearly identical to the *suppression* variant (see Figure S10).

For the *suppression* and *combined* DBS variants, the *history* factor (DBS ON vs. OFF) showed no significant main effect [two‐way repeated measures ANOVA, within factor history (ON, OFF), *suppression*: *F*(1, 99) < 0.001, *p* = 0.982, *combined*: *F*(1, 99) = 0.266, *p* = 0.607]. In contrast, applying DBS during the trials (*acute* factor) had a significant main effect, indicating that DBS primarily influences behavior through acute activity changes [within factor acute (ON, OFF), *suppression*: *F*(1, 99) = 232,893, *p* < 0.001, *combined*: *F*(1, 99) = 177,388, *p* < 0.001].

However, a strong interaction between the *history* and *acute* factors was observed [*suppression*: *F*(1, 99) = 232,893, *p* < 0.001, *combined*: *F*(1, 99) = 177,388, *p* < 0.001]. Post hoc *t*‐tests revealed that applying DBS during trials (acute = ON) had no effect when the model states had a DBS OFF history. Only applying DBS in model states with a DBS ON history significantly increased the number of unrewarded decisions [paired *t*‐test, *suppression*: *t*(99) = −15.261, *p* < 0.001, *combined*: *t*(99) = −13.319, *p* < 0.001]. This suggests that DBS needs to first modulate plasticity before acute activity changes can impact behavior.

Further post hoc *t*‐tests showed that, when DBS was applied during trials (acute = ON), a DBS ON history resulted in significantly more unrewarded decisions compared with a DBS OFF history [paired *t*‐test, *suppression*: *t*(99) = −4.772, *p* < 0.001, *combined*: *t*(99) = −4.994, *p* < 0.001]. Conversely, when DBS was not applied during trials (acute = OFF), a DBS ON history resulted in significantly fewer unrewarded decisions compared with a DBS OFF history [paired *t*‐test, *suppression*: *t*(99) = 5.572, *p* < 0.001, *combined*: *t*(99) = 4.932, *p* < 0.001]. These findings indicate that the behavioral effects of plasticity modulation are highly dependent on whether DBS is currently active. Interestingly, plasticity modulation alone, without acute activity changes (as in the case of sudden DBS deactivation), produced the opposite effect on behavior compared with continuous DBS application.

For the *efferent* DBS variant, the model showed significantly more unrewarded decisions in model states with a DBS ON history compared with those with a DBS OFF history [two‐way repeated measures ANOVA, within factor history (ON, OFF), *efferent*: *F*(1, 99) = 14.243, *p* < 0.001]. Applying DBS during the trials had no additional effect, indicating that the efferent variant influences behavior exclusively through plasticity modulation. Unlike the suppression and combined variants, the behavioral effects of the efferent variant did not reverse when DBS was suddenly turned off. This aligns with our other findings, where the *efferent* variant consistently exhibits distinct patterns of activity and plasticity modulation compared with the *suppression* and *combined* variants (see Sections 3.4 and 3.5).

In summary, the *suppression* and *combined* variants influence behavior through a combination of plasticity modulation and acute activity changes, whereas the *efferent* variant operates exclusively through plasticity modulation. This distinction underscores unique mechanisms by which potential different DBS variants may affect decision‐making behavior. Notably, it highlights a potentially testable difference among DBS variants: their varying effects on behavior following the abrupt deactivation of DBS.

### 3.8 Parameter Search for DBS Variants

**FIGURE 12:**
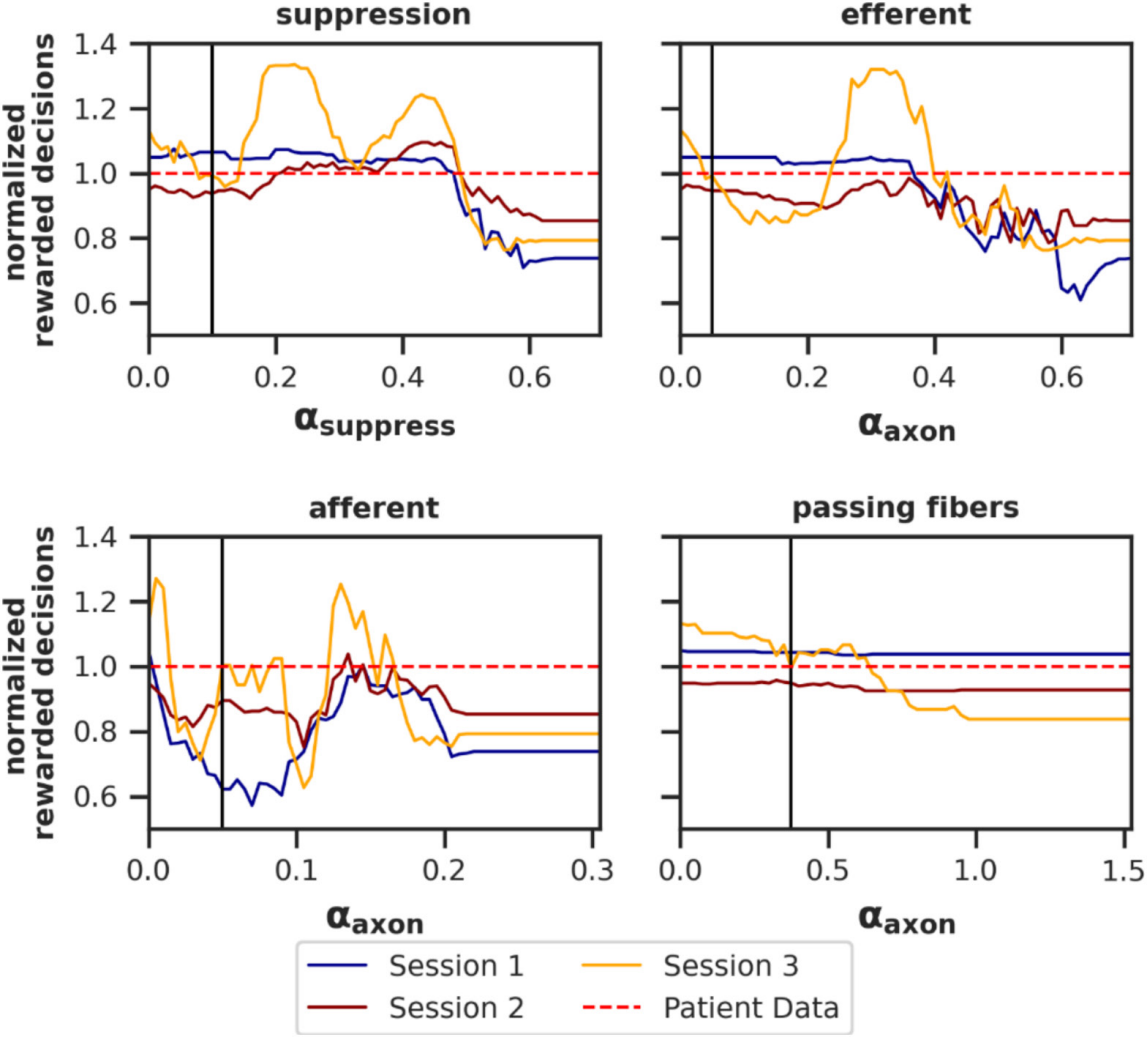
Normalized number of rewarded decisions in the three sessions of the task for various parameter sets of the different DBS variants. Data for each session are normalized by the patients' data of the corresponding session (dashed red line). The lines show the average over 14 simulations/patients. The vertical black line shows the final selected parameter values for the DBS variants, which led to the smallest average difference between the model's and patients' data (mean and variance) over all three sessions. For the *afferent* DBS variant, the same parameter value as for the *efferent* variant is marked because no suitable parameter value for *afferent* could be found.

The reversal learning task was simulated using various parameter values for each DBS variant. Parameters were selected to minimize the mean deviation between the model's performance (number of rewarded decisions per session) and patient data in the DBS ON state. Figure 12 illustrates the model's performance relative to patient performance across tested DBS variant parameters.

When multiple parameter values showed similarly low deviations from the patient data mean values, those that best captured the data variance were chosen. For the *afferent* DBS variant, no suitable parameter values were found due to significant fluctuations in mean performance and high variance of the data. Consequently, the *afferent* DBS variant was excluded from further reversal learning task simulations. For analyzing the effects of DBS on population activities (Section 3.4) and plasticity of projections (Section 3.5), the same parameter was used for the *afferent* variant as for the *efferent* variant. For the *combined* variant, the parameter values from the individual DBS variants (excluding *afferent*) were slightly adjusted manually to align with patient data.

## 4 Discussion

Our study aims to explore by means of a neuro‐computational model how DBS within the globus pallidus internus (GPi) influences decision‐making processes in the basal ganglia, particularly in the context of a probabilistic reversal learning task. We specifically investigated whether DBS promotes habitual biases in decision‐making by enhancing the influence of a cortico‐thalamic shortcut and, therefore, reducing the basal ganglia's role in decision‐making. Our findings show that DBS in our model indeed shifts the balance toward cortico‐thalamic‐driven habitual our through both acute activity changes and modulated plasticity within the basal ganglia circuitry.

### 4.1 DBS and Habitual Biases

Consistent with our previous work (Baladron and Hamker 2020; Scholl et al. 2022), our simulations demonstrate that a slow learning cortico‐thalamic shortcut bypassing the basal ganglia contributes to the formation of habit behavior. The gradually evolving bias toward habitual behavior caused by the learning shortcut leads to increased numbers of unrewarded decisions after reward reversal in the simulated reversal learning task, which is required to replicate the patient data from de A Marcelino et al. (2023). Applying DBS within the GPi further increases the number of unrewarded decisions after reversal. Recordings of thalamic inputs in the model support our hypothesis that DBS within the GPi reduces the influence of the basal ganglia, thereby amplifying the cortico‐thalamic shortcut's role in decision‐making and enhancing habitual biases in decision‐making.

Usually, habits need to be tested with outcome devaluation tasks (de Wit et al. 2018). Reversal learning measures the disengagement from ongoing responses (Izquierdo et al. 2017) and is thus not a typical habitual learning task. However, we previously discussed the role of cortico‐thalamic pathways that bypass the basal ganglia (Schroll et al. 2014) and proposed that the interaction between fast learners (the basal ganglia) and slow learners (cortico‐thalamic and cortico‐cortical shortcuts) represents a general functional principle of the brain (Baladron and Hamker 2020; Maith, Schwarz, and Hamker 2021; Villagrasa et al. 2018). We propose that these gradual learning mechanisms already cause habitual biases in decision‐making before actual habit behavior manifests.

Our model does not specify particular cortical regions. The findings of Smith and Graybiel (2013) highlight the infralimbic cortex, which is part of the prefrontal cortex, as playing a key role in habit formation, suggesting its potential involvement in such shortcuts. Given that the task in our study involves visual decision‐making, it is reasonable to hypothesize that a shortcut between the inferior temporal cortex and the prefrontal cortex is trained via an open visual cortico‐striatal loop (Seger 2013).

Four out of the five tested DBS variants (*suppression*, *efferent*, *passing fibers*, and *combined*) can replicate the patient data from de A Marcelino et al. (2023) to varying extents. This suggests that multiple pathways can lead to similar behavioral outcomes, underscoring the complexity of DBS's effects on basal ganglia circuitry. Specifically, the *suppression*, *efferent*, and *combined* DBS variants show significant increases in unrewarded decisions in trials after reward reversal, supporting our hypothesis. We demonstrated that the behavioral effects of these DBS variants result from a combination of activity and plasticity modulation. Notably, the DBS variants exhibit distinct behavioral effects after stimulation is switched off. This observation could be further explored experimentally to pinpoint the specific DBS mechanisms that influence behavior. This, in turn, emphasizes the usefulness of computational models in making testable predictions and model‐based data analysis.

de A Marcelino et al. (2023) effectively replicated human participants' choices and response times in the reversal learning task using a reinforcement learning drift diffusion model (RLDDM). Based on their fitted RLDDM, they propose that DBS leads to increased exploratory behavior, as indicated by a higher proportion of low‐value choices according to the RLDDM choice values. Similarly, we fitted a temporal difference (TD) learning model with softmax action selection to the patients' and model's data. Although our model exhibits an increased habitual bias in decision‐making, we also observed a higher proportion of low‐value choices caused by DBS. This indicates that the approach used to estimate exploratory choices in this two‐choice reward reversal learning task may not effectively distinguish between exploratory and habitual behaviors. While exploratory behavior promotes random choices, habitual behavior promotes previously rewarded decisions. Both can lead to a decreased tendency to select the option with the highest value according to the TD algorithm. Moreover, unlike RLDDM or TD model approaches, our neuro‐computational model enables the inference of specific mechanisms within the basal ganglia that drive the observed behavior and allows for the direct implementation of potential neuronal mechanisms underlying DBS effects.

### 4.2 Mechanisms of DBS Action

The different DBS variants have distinct effects on the activity of neural populations within the model. For example, the *suppression* variant inhibits GPi neurons, leading to increased thalamic and cortical (Cor_dec_) activity, while the *efferent* variant increases GPi output, also inhibiting the GPi but reducing thalamic and cortical activity. Despite these differences in neural activity patterns, both variants result in similar behavioral outcomes, indicating that multiple pathways can lead to the same functional effect.

A common hypothesis is that the primary mechanism by which DBS influences behavior is through the inhibition of stimulated neurons (Benazzouz and Hallett 2000; Beurrier et al. 2001; Boraud et al. 1996; Dostrovsky et al. 2000; Meissner et al. 2005). Our results also show that the inhibition of the GPi is a common feature of the different tested DBS variants. However, the lack of a simple relationship between GPi activity and the number of rewarded decisions indicates that other factors, such as the stimulation of axons, which do not solely inhibit the GPi, and the dynamics of neural plasticity, also play critical roles. This aligns with the more recent view that DBS effects on the network level are much more complex, primarily by stimulating axons separately from the soma, affecting both nearby and distant neuronal structures in various ways (McIntyre and Hahn 2010; Neumann, Steiner, and Milosevic 2023).

### 4.3 Plasticity and Acute Effects of DBS

Numerous studies have demonstrated that, in addition to causing acute activity changes, DBS also affects synaptic plasticity within the basal ganglia (Dvorzhak et al. 2013; Erez et al. 2009; Milosevic et al. 2019; Shen et al. 2003; Steiner et al. 2019; van Hartevelt et al. 2014). Thus, the interaction between DBS‐induced acute activity changes and plasticity modulation is crucial for understanding its functional effects on the basal ganglia circuitry. Our simulations revealed that the acute activity changes induced by DBS are necessary but not sufficient on their own to replicate the patient data. Instead, the modulation of plasticity by DBS was necessary for the observed behavioral effects, suggesting that DBS influences learning processes within the basal ganglia. Furthermore, the modulated plasticity seems to learn a compensation for the DBS‐induced activity changes for the *suppression* DBS variant.

Interestingly, the *efferent* DBS variant influenced behavior exclusively through changes in plasticity, with its effects persisting even after the cessation of acute DBS‐induced activity changes. This highlights that different DBS effects on the nearby tissue might affect the functional circuitry and behavior differently in DBS ON and OFF phases, which might be an interesting marker for determining which effects on the nearbsy tissue drive behavior.

### 4.4 Implications for DBS as a Treatment

Our findings have some implications for the clinical use of DBS in treating neurological conditions. Understanding that DBS in the basal ganglia can shift the balance toward habitual behavior suggests that treatment protocols could be optimized to enhance or diminish this effect, depending on the desired therapeutic outcome. For instance, in conditions where habitual behavior is detrimental, such as Tourette syndrome (Delorme et al. 2016; Scholl et al. 2022), DBS protocols might be adjusted to minimize the enhancement of cortico‐thalamic or cortico‐cortical shortcuts bypassing the basal ganglia.

Additionally, our study underscores the importance of tailored DBS configurations. Considering that the effects of DBS on nearby tissue can vary depending on the electrode placement and underlying anatomy, it is crucial to recognize that these diverse effects also result in distinct behavioral modulations, as shown here. Given that these different effects also differently modulate plasticity and show different behavioral modulations after the cessation of acute DBS‐induced activity changes, it may be helpful to carefully monitor the behavior after turning DBS OFF to elucidate the underlying mechanisms that influence behavior.

### 4.5 Limitations

While our model successfully replicates key aspects of patient data, it has certain limitations. First, its simplifications, such as the use of artificial rate‐coded neurons and a simplified structure, do not fully capture the complexity of the biological system. Notably, interactions with other brain regions are omitted, despite findings by de A Marcelino et al. (2023) showing that the extent of DBS‐induced exploration is associated with the functional connectivity between the stimulation electrode site and a distributed brain network. Additionally, the local striatal microcircuit is highly complex, integrating inputs from nearly all cortical and various subcortical areas through diverse interneuron subtypes (Hjorth et al. 2020; Johansson and Silberberg 2020). Our model abstracts away this complexity to focus on broader system‐level interactions within the basal ganglia and cortico‐thalamic pathways.

Fast‐spiking interneurons (FSIs), which play a crucial role in striatal microcircuits by contributing to action initiation (Berke 2011) and habit formation (O'Hare et al. 2017), are not explicitly modeled. However, a simple winner‐takes‐all mechanism that FSIs may similarly facilitate (O'Hare et al. 2017) is represented in our model through lateral inhibition between projection neurons. Importantly, our core hypothesis, that DBS in the GPi reduces basal ganglia influence and shifts decision‐making toward the cortico‐thalamic pathway, remains compatible with the role of FSIs in habit formation. The habit‐related effects of FSIs (O'Hare et al. 2017) could likely occur in parallel with cortico‐thalamic learning and do not contradict our model's conclusions.

Although the model's structure is significantly simplified and, unlike other models (Goenner et al. 2021; Hjorth et al. 2020), its artificial firing rates only indirectly correspond to empirical data, it uniquely demonstrates how the functional aspects of the basal ganglia emerge through learning, an aspect often not captured by other models. This capability enables the simulation of learning tasks, such as those described by de A Marcelino et al. (2023). Instead of specifying synaptic connectivity patterns or strength within the basal ganglia pathways, our model includes mechanisms through which connectivity self‐organizes via synaptic plasticity. Consequently, the pathways do not serve any predefined function but develop their roles by adjusting synaptic weights (Schroll et al. 2014).

The inclusion of dopamine‐modulated synaptic plasticity in extrastriatal connections within our model remains an assumption based on indirect experimental evidence. While direct evidence for such plasticity is lacking, three main lines of research support its plausibility: (1) anatomical studies demonstrate that the subthalamic nucleus (STN), globus pallidus externus (GPe), and globus pallidus internus (GPi) receive dopaminergic innervation from the substantia nigra pars compacta (SNc) (Cossette et al. 1999; Dong et al. 2021; Gauthier et al. 1999; Prensa et al. 2000; Rajput et al. 2008); (2) dopamine receptors, including both D1 and D2 subtypes, have been identified in these regions (Boyson et al. 1986; Flores et al. 1999; Levey et al. 1993); and (3) functional studies suggest that dopamine influences synaptic transmission and neural dynamics in these areas (Bouali‐Benazzouz et al. 2009; Dong et al. 2021; Prescott et al. 2009). Despite this indirect support, further experimental validation is necessary to confirm dopamine‐modulated plasticity in these extrastriatal connections.

Our study focused on a specific task and set of DBS parameters. Expanding the range of tasks and exploring different parameter settings could provide a more comprehensive understanding of DBS's effects across various contexts and conditions. Additionally, investigating the role of other basal ganglia structures and pathways not included in our model, such as the recently discovered distinct GPe neuron subtypes (Abdi et al. 2015; Gittis et al. 2014; Mallet et al. 2012, 2016), could further elucidate the network‐wide effects of DBS. Moreover, experimental validation of our computational findings in animal models and clinical studies is essential.

Although our results support our hypothesis that GPi DBS promotes habit behavior, it is important to acknowledge that we cannot rule out the hypothesis of de A Marcelino et al. (2023), suggesting that GPi DBS promotes exploratory behavior. Both models explain the patient data and align with current theories regarding the effects of DBS, which include the inhibition and informational lesion of the region. Further experimental research is needed to differentiate these possible explanations more precisely. One potential approach could be a multiple‐choice reversal learning task (Maith et al. 2023), which could more effectively distinguish between habitual behavior (selecting the action most frequently rewarded before the reversal) and exploration (selecting alternative actions) after a reversal.

## 5 Conclusion

In summary, our study offers insights into how DBS within the GPi influences decision‐making and habitual behavior through a combination of acute neuronal activity changes and modulated synaptic plasticity. Further, our findings enhance our understanding of the mechanisms of action underlying DBS. Our work suggests that the influence of cortico‐thalamic or cortico‐cortical shortcuts, bypassing the basal ganglia, on decision‐making may be enhanced by DBS in the basal ganglia. We demonstrate that habitual behavior can overlap with exploratory behavior in a two‐choice reversal learning task, thus, future studies should employ tasks that clearly differentiate between the two.

## Author Contributions

**Oliver Maith:** data curation, formal analysis, funding acquisition, methodology, project administration, software, supervision, validation, visualization, writing – original draft, writing – review and editing. **Dave Apenburg:** data curation, formal analysis, investigation, methodology, software, validation, visualization, writing – original draft. **Fred Hamker:** conceptualization, funding acquisition, project administration, resources, supervision, writing – review and editing.

## Conflicts of Interest

The authors declare no conflicts of interest.

### Peer Review

The peer review history for this article is available at https://www.webofscience.com/api/gateway/wos/peer‐review/10.1111/ejn.70130.

## Supporting information


**Figure S1.** Related to Figure 2 in the main article. The number of unrewarded decisions throughout the task in 5‐trial bins averaged over the 14 patients from de A Marcelino et al. (2023) in the DBS OFF condition and 14 simulations with the model with plastic and fixed cortico‐thalamic shortcut. A prominent temporary increase in unrewarded decisions can be seen right after the reward reversal at trial 60.
**Figure S2.** Related to Figure 3 in the main article. Comparison of unrewarded decisions between patients from de A Marcelino et al. (2023) and simulations for all DBS variants (except afferent) for the model with the plastic shortcut. Significant differences between patient data and simulations, as identified by post hoc *t*‐tests, are annotated. A – suppression, B – efferent, C – passing fibers, D – combined. All DBS variants show very similar patterns for the unrewarded decisions. The data of 14 patients / 100 simulations are displayed as boxplots: horizontal line ‐ the median, box – the interquartile range (IQR) from the 25th percentile to the 75th percentile, whiskers – extending up to 1.5 times the IQR, circles – outliers outside 1.5 times the IQR.
**Figure S3.** Related to Figure 3 in the main article. Comparison of unrewarded decisions between patients from de A Marcelino et al. (2023) and simulations for all DBS variants (except afferent) for the model with the fixed shortcut. Significant differences between patient data and simulations, as identified by post hoc *t*‐tests, are annotated. A – suppression, B – efferent, C – passing fibers, D – combined. All DBS variants show very similar patterns for the unrewarded decisions. The data of 14 patients/100 simulations are displayed as boxplots: horizontal line ‐ the median, box – the interquartile range (IQR) from the 25th percentile to the 75th percentile, whiskers – extending up to 1.5 times the IQR, circles – outliers outside 1.5 times the IQR.
**Figure S4.** Model comparison between the single rate and dual rate model in which the dual rate model is ranked better (highlighted with vertical dashed line). elpd loo – expected log pointwise predictive density leave‐one‐out cross‐validation (Vehtari et al., 2017)
**Figure S5.** Related to Figure 4 of the main article. Average activities of the populations of the untrained model in the DBS OFF condition. Bar lengths show the average over 100 simulations with error bars showing the SD. Each simulation consisted of a single trial, thus, simulating the untrained model, where the rates were calculated from the time window 2500–3000 ms.
**Figure S6.** Related to Figure 8 and Figure 12 of the main article. Normalized number of rewarded decisions averaged over the three sessions of the task for various parameter sets of the simulated DBS variants (orange line) together with the average rate of the GPi population (blue line) obtained from single trials performed by the untrained model. The number of rewarded decisions for each session is normalized by the patients’ data of the corresponding session (dashed red line). The data are averaged over 14 simulations/patients.
**Figure S7.** Related to Figure 9 in the main article. Average calculated weights from the Cor_in_ to the two output neurons in Cor_dec_. Each row displays a different plastic pathway and each column a different DBS variant. The vertical lines indicate the session boundaries. The weights are averaged over 100 simulations (in each DBS condition) the transparent area displays the 95% confidence interval. Above the x‐axis significant differences between DBS ON and OFF are indicated for the output 0 in black and output 1 in gray. P‐values were corrected for multiple comparisons using the Benjamini‐Hochberg procedure (Benjamini & Hochberg, 1995).
**Figure S8.** Related to Figure 10 in the main article. Support of the shortcut and basal ganglia in the thalamus for the initially frequently rewarded option throughout the task in 5‐trial bins. A – DBS OFF, B – suppression, C – efferent, D – combined. Notably, basal ganglia support quickly adapts after the reversal at trial 60 while the shortcut support very slowly adapts and still supports the initially frequently rewarded option after the reversal. The lines show the average over 100 simulations with the transparent area representing the 95% confidence interval.
**Figure S9.** Related to Figure 10 in the main article. Support of the shortcut and basal ganglia in the thalamus for the selected option for the model with the fixed shortcut. Significant DBS effects (compared to DBS OFF) are annotated. Notably, the decrease of the basal ganglia support induced by DBS is very small in the model with the fixed shortcut. The data of 100 simulations are displayed as boxplots: horizontal line ‐ the median, triangle – the mean, box – the interquartile range (IQR) from the 25^th^ percentile to the 75^th^ percentile, whiskers – extending up to 1.5 times the IQR, circles – outliers outside 1.5 times the IQR.
**Figure S10.** Related to Figure 11 in the main article. Number of unrewarded decisions in the third session of the task depending on whether DBS is applied during trials (acute) and whether DBS was applied in all trials before (history). Significant effects of acute and history, as identified by post hoc *t*‐tests, are annotated, for acute below the boxplots and for history above the boxplots. Exclusively applying DBS during trials (acute = on) without applying it in all trials before (history = off) does not lead to any behavioral changes (compared to both off). The DBS variants suppression and combined shown here have almost identical effects on unrewarded decisions. The data of 100 simulations are displayed as boxplots: horizontal line ‐ the median, box – the interquartile range (IQR) from the 25th percentile to the 75th percentile, whiskers – extending up to 1.5 times the IQR, circles – outliers outside 1.5 times the IQR.
**Table S1.** Parameters of dopamine‐modulated learning in cortical input projections
**Table S2.** Parameters of dopamine‐modulated learning in projections within the basal ganglia
**Table S3.** Population parameters of the model from Maith et al. (2021).
**Table S4.** Connection types and weights of the projections of the model from Maith et al. (2021).
**Table S5.** Shortcut projection’s plasticity parameters of the model from Maith et al. (2021)
**Table S6.** Parameters of dopamine‐modulated learning in cortical input projections of the model from Maith et al. (2021)
**Table S7.** Parameters of dopamine‐modulated learning in projections within the basal ganglia of the model from Maith et al. (2021)
**Table S8.** Differences in firing rates compared to DBS OFF for all DBS variants
**Table S9.** Firing rates of the model for DBS OFF and all DBS variants

## Data Availability

The simulation data generated in this study have been deposited at Zenodo and are publicly available as of the date of publication at 10.5281/zenodo.12819011. All original code has been deposited at Zenodo and is publicly available as of the date of publication at https://zenodo.org/doi/10.5281/zenodo.12819164.
