# Supplementary material for: Pallidal Deep Brain Stimulation Enhances Habitual Behavior in a Neuro‐Computational Basal Ganglia Model During a Reward Reversal Learning Task
Source: Eur J Neurosci. 2025 May 5;61(9):e70130. doi: 10.1111/ejn.70130 (PMC12053244; doi:10.1111/ejn.70130)
Supplement: Supplementary file 1 — Figure S1. Related to Figure 2 in the main article. The number of unrewarded decisions throughout the task in 5‐trial bins averaged over the 14 patients from de A Marcelino et al. (2023) in the DBS OFF condition and 14 simulations with the model with plastic and fixed cortico‐thalamic shortcut. A prominent temporary increase in unrewarded decisions can be seen right after the reward reversal at trial 60. Figure S2. Related to Figure 3 in the main article. Comparison of unrewarded decisions between patients from de A Marcelino et al. (2023) and simulations for all DBS variants (except afferent) for the model with the plastic shortcut. Significant differences between patient data and simulations, as identified by post hoc t‐tests, are annotated. A – suppression, B – efferent, C – passing fibers, D – combined. All DBS variants show very similar patterns for the unrewarded decisions. The data of 14 patients / 100 simulations are displayed as boxplots: horizontal line ‐ the median, box – the interquartile range (IQR) from the 25th percentile to the 75th percentile, whiskers – extending up to 1.5 times the IQR, circles – outliers outside 1.5 times the IQR. Figure S3. Related to Figure 3 in the main article. Comparison of unrewarded decisions between patients from de A Marcelino et al. (2023) and simulations for all DBS variants (except afferent) for the model with the fixed shortcut. Significant differences between patient data and simulations, as identified by post hoc t‐tests, are annotated. A – suppression, B – efferent, C – passing fibers, D – combined. All DBS variants show very similar patterns for the unrewarded decisions. The data of 14 patients/100 simulations are displayed as boxplots: horizontal line ‐ the median, box – the interquartile range (IQR) from the 25th percentile to the 75th percentile, whiskers – extending up to 1.5 times the IQR, circles – outliers outside 1.5 times the IQR. Figure S4. Model comparison between the single rate and dual rate model in [file EJN-61-0-s001.docx]

## Dopamine-modulated learning in the model

The dopamine-modulated plasticity mechanisms were adopted directly from Maith et al. (2021), with minor parameter adjustments (see Section **S2**). While we include the equations here, we refer readers to Schroll et al. (2014) and Maith et al. (2021) for a more detailed description of the functional effects of this implementation.

The plasticity in the projections from Cor_in_ to StrD1, StrD2 and STN (i.e., the direct, indirect and hyperdirect pathway) is described by a dopamine-modulated learning rule defined by the following equations:

| $\tau_{w}\frac{dw}{dt}=\alpha_{dopa}*r_{pre}v- \alpha v^{2}$ | (S1.1) |
| --- | --- |
| $\tau_{\alpha}\frac{d\alpha}{dt}={({mp}_{post}-\theta_{reg})}^{+}-\alpha$ | (S1.2) |
| $v={(r_{post}-\bar{r}_{post})}^{+}$ | (S1.3) |
| $\alpha_{dopa}=\delta_{dopa}\left\{ \begin{aligned} \gamma_{burst}, &\delta_{dopa}>0 \\ \gamma_{dip}, &\delta_{dopa}\leq0 \end{aligned} \right.$ | (S1.4) |
| $\delta_{dopa}=\beta\left( r_{SNc}-B_{SNc} \right)$ | (S1.5) |

This rule involves the weight $w$, a regularization variable $\alpha$, pre- and postsynaptic rates $r_{pre}$ and $r_{post}$, the population average of the postsynaptic rate $\bar{r}_{post}$, the postsynaptic membrane potential ${mp}_{post}$, the rate $r_{SNc}$ and baseline $B_{SNc}$ of the SNc neuron representing changes in dopamine. The corresponding parameters are described in Table **S1**.

The projections of the direct (StrD1-GPi, Equations **S2.1-S2.7**), indirect (StrD2-GPe, Equations **S3.1-S3.2**), and hyperdirect (STN-GPi, Equations **S4.1-S4.3**) pathways within the basal ganglia also undergo dopamine-modulated plasticity. This plasticity differs from that of cortical inputs primarily because, in addition to excitatory projections, the basal ganglia include inhibitory projections (originating from the striatum) and tonically active populations (GPe, GPi, STN). The plasticity is described by the following equations:

| $\tau_{w}\frac{dw}{dt}=\alpha_{dopa}*uv- \alpha{(uv)}^{+}$ | (S2.1) |
| --- | --- |
| $\tau_{\alpha}\frac{d\alpha}{dt}={({-mp}_{post}-\theta_{reg})}^{+}-\alpha$ | (S2.2) |
| $u={(r_{pre}-\text{mean}\left( r_{pre} \right))}^{+}$ | (S2.3) |
| $v=-r_{post}+\left\{ \begin{aligned} \text{min(}r_{post})+\gamma, &\text{min(}r_{post})+\gamma<\text{mean}\left( r_{post} \right)-\theta_{post} \\ \text{mean}\left( r_{post} \right)-\theta_{post}, &\text{min(}r_{post})+\gamma\geq\text{mean}\left( r_{post} \right)-\theta_{post} \end{aligned} \right.$ | (S2.4) |
| $\alpha_{dopa}=\delta_{dopa}\left\{ \begin{aligned} \gamma_{burst} \left( \varepsilon+\varphi\left( 1-\varepsilon\right) \right), &\delta_{dopa}>0 \\ \gamma_{dip} \varepsilon, &\delta_{dopa}\leq0 \end{aligned} \right.$ | (S2.5) |
| $\delta_{dopa}=r_{SNc}-B_{SNc}$ | (S2.6) |
| $\varepsilon=\left\{ \begin{aligned} 1, &v>0 \\ 0, &v\leq0 \end{aligned} \right.$ | (S2.7) |

| $v=\text{max}\left( r_{post} \right)-\theta_{post}-r_{post}$ | (S3.1) |
| --- | --- |
| $\delta_{dopa}=-\left( r_{SNc}-B_{SNc} \right)$ | (S3.2) |

| $\tau_{\alpha}\frac{d\alpha}{dt}={({mp}_{post}-\theta_{reg})}^{+}-\alpha$ | (S4.1) |
| --- | --- |
| $v=r_{post}-\left\{ \begin{aligned} \text{min(}r_{post})+\gamma, &\text{min(}r_{post})+\gamma<\text{mean}\left( r_{post} \right)+\theta_{post} \\ \text{mean}\left( r_{post} \right)+\theta_{post}, &\text{min(}r_{post})+\gamma\geq\text{mean}\left( r_{post} \right)+\theta_{post} \end{aligned} \right.$ | (S4.2) |
| $\varepsilon=\left\{ \begin{aligned} 1, &v<0 \\ 0, &v\geq0 \end{aligned} \right.$ | (S4.3) |

For the indirect and hyperdirect pathways only the equations which differ from **Equations S2.1-S2.7** are shown (otherwise they are the same). These rules involve the weight $w$, a regularization variable $\alpha$, pre- and postsynaptic rates $r_{pre}$ and $r_{post}$, the population minimum (min), maximum (max) and average (mean) of the rates, the postsynaptic membrane potential ${mp}_{post}$, the rate $r_{SNc}$ and baseline $B_{SNc}$ of the SNc neuron representing changes in dopamine. The corresponding parameters are described in Table **S2**.

The plasticity in the projection from StrD1 to SNc represents a reward prediction causing weights to increase upon repeated reward. The corresponding learning rule is described by the following equations:

| $\tau_{w}\frac{dw}{dt}=uv$ | (S5.1) |
| --- | --- |
| $\tau_{w}=\left\{ \begin{aligned} 300, &r_{PPN}>0 \\ 100, &r_{PPN}\leq0 \end{aligned} \right.$ | (S5.2) |
| $u={{(r}_{StrD1}-\bar{r}_{StrD1})}^{+}$ | (S5.3) |
| $v=(r_{SNc}-B_{SNc})$ | (S5.4) |

with the weight $w$, time constant $\tau_{w}$, the rates of StrD1 $r_{StrD1}$ and their population average $\bar{r}_{StrD1}$, the rate of the PPN neuron $r_{PPN}$, and the rate $r_{SNc}$ and baseline $B_{SNc}$ of the SNc neuron representing changes in dopamine.

## Parameter adjustments to replicate DBS OFF patient data

We adjusted the basal ganglia model from Maith et al. (2021) to simulate the reversal learning task described by de A Marcelino et al. (2023). In general, the parameters were determined to guarantee the function of the model. The model was manually tuned in multiple steps. First, the reversal learning task was simulated with 100% reward probabilities, which were then changed to 80:20. To evaluate the model's performance and compare it with the behavioral data from de A Marcelino et al. (2023), we ran 14 simulations for each tested parameter set.

The main changes implemented are as follows: a plastic cortico-thalamic shortcut projection replaced the plastic cortico-cortical shortcut projection; the population dimensions were reduced since the previous model encoded 16 outputs instead of 2; the GPi lateral connections are now inhibitory; and the learning speed of the plastic projections was adjusted. To easily track these changes, all parameters from Maith et al. (2021) are detailed in Table **S3** - Table **S7**.

## Supplementary Figures


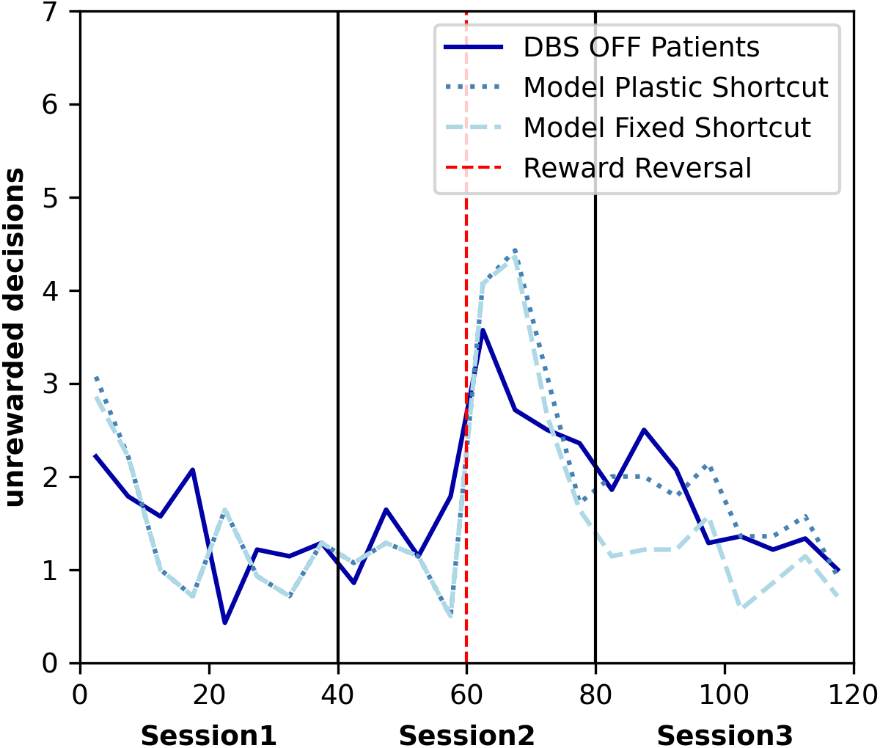


**Figure S1** Related to Figure **2** in the main article. The number of unrewarded decisions throughout the task in 5-trial bins averaged over the 14 patients from de A Marcelino et al. (2023) in the DBS OFF condition and 14 simulations with the model with plastic and fixed cortico-thalamic shortcut. A prominent temporary increase in unrewarded decisions can be seen right after the reward reversal at trial 60.


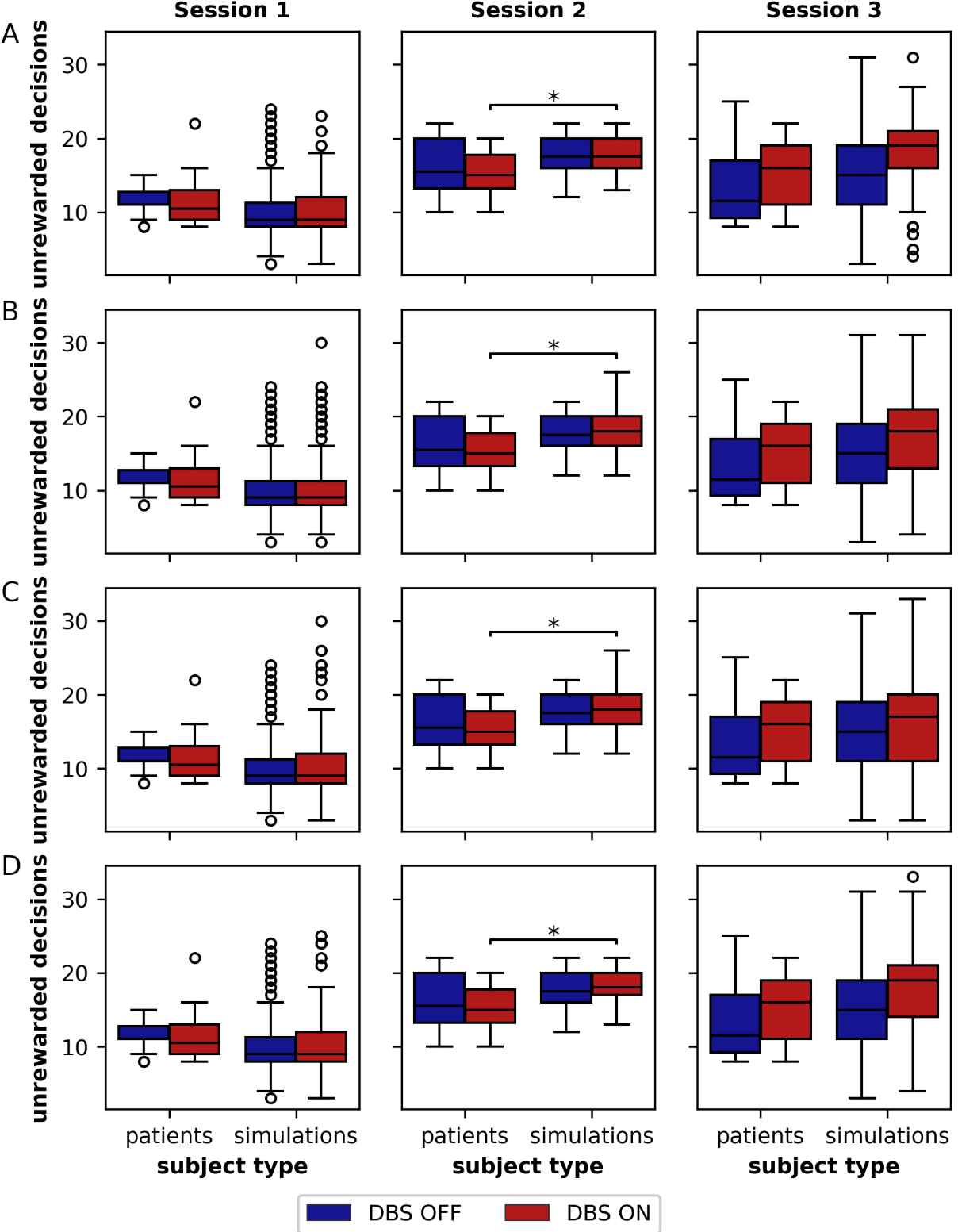


**Figure S2** Related to Figure **3** in the main article. Comparison of unrewarded decisions between patients from de A Marcelino et al. (2023) and simulations for all DBS variants (except afferent) for the model with the plastic shortcut. Significant differences between patient data and simulations, as identified by post-hoc t-tests, are annotated. A – suppression, B – efferent, C – passing fibers, D – combined. All DBS variants show very similiar patterns for the unrewarded decisions. The data of 14 patients / 100 simulations are displayed as boxplots: horizontal line - the median, box – the interquartile range (IQR) from the 25th percentile to the 75th percentile, whiskers – extending up to 1.5 times the IQR, circles – outliers outside 1.5 times the IQR.


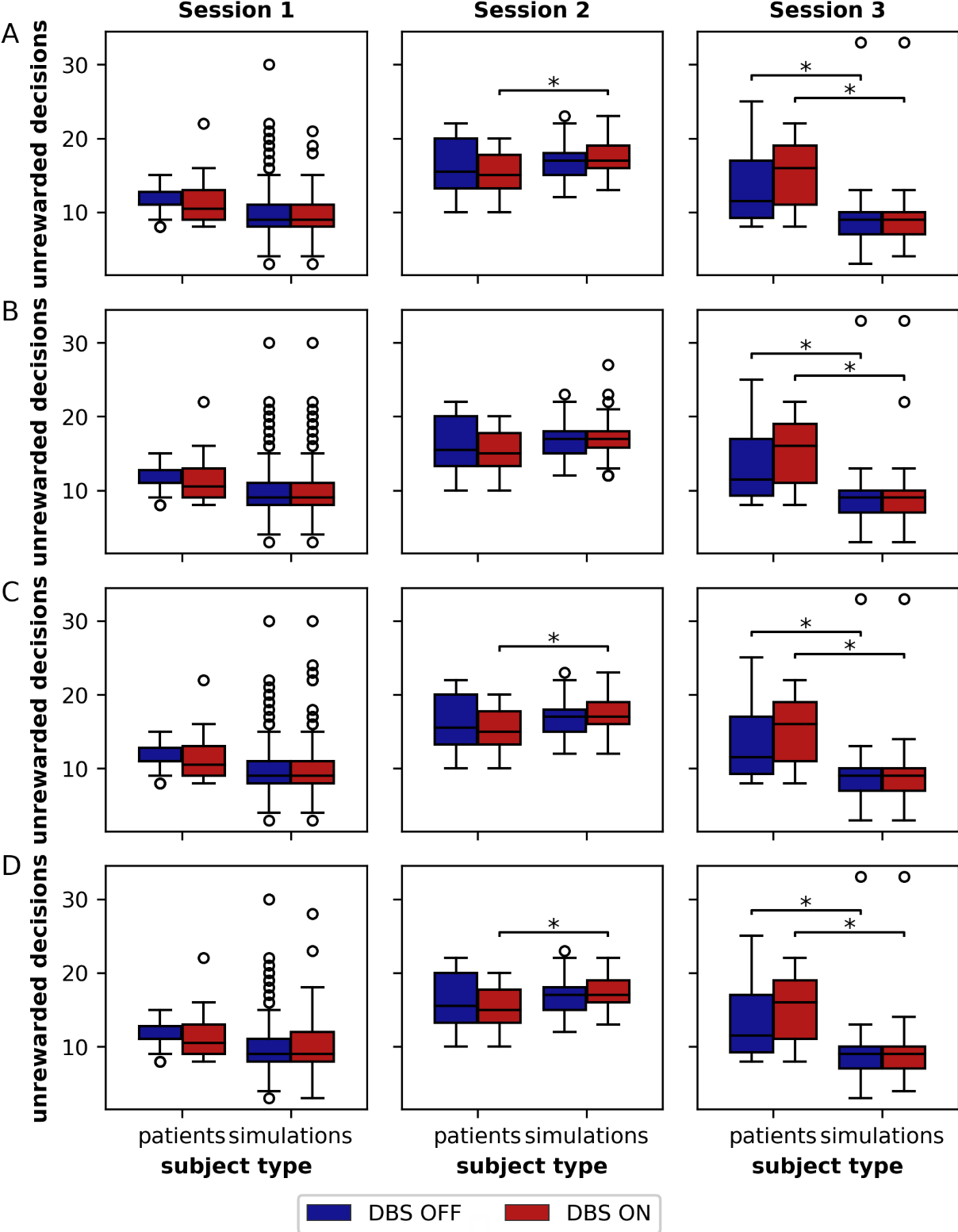


**Figure S3** Related to Figure **3** in the main article. Comparison of unrewarded decisions between patients from de A Marcelino et al. (2023) and simulations for all DBS variants (except afferent) for the model with the fixed shortcut. Significant differences between patient data and simulations, as identified by post-hoc t-tests, are annotated. A – suppression, B – efferent, C – passing fibers, D – combined. All DBS variants show very similiar patterns for the unrewarded decisions. The data of 14 patients / 100 simulations are displayed as boxplots: horizontal line - the median, box – the interquartile range (IQR) from the 25th percentile to the 75th percentile, whiskers – extending up to 1.5 times the IQR, circles – outliers outside 1.5 times the IQR.


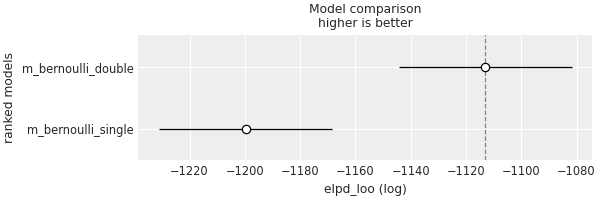


**Figure S4** Model comparison between the single rate and dual rate model in which the dual rate model is ranked better (highlighted with vertical dashed line). elpd loo – expected log pointwise predictive density leave-one-out cross-validation (Vehtari et al., 2017)


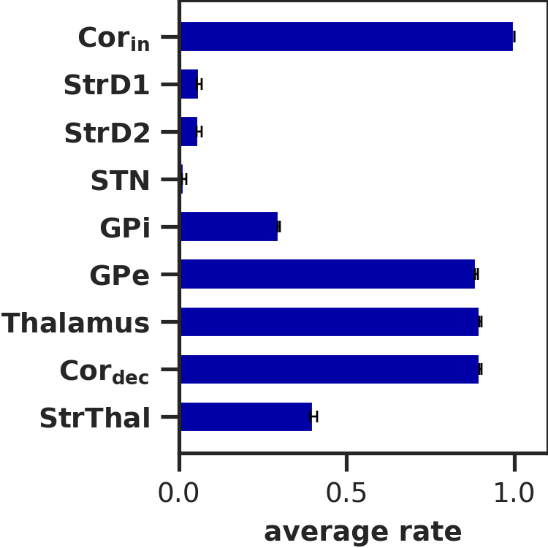


**Figure S5** Related to Figure **4** of the main article. Average activities of the populations of the untrained model in the DBS OFF condition. Bar lengths show the average over 100 simulations with error bars showing the SD. Each simulation consisted of a single trial, thus, simulating the untrained model, where the rates were calculated from the time window 2500 – 3000 ms.


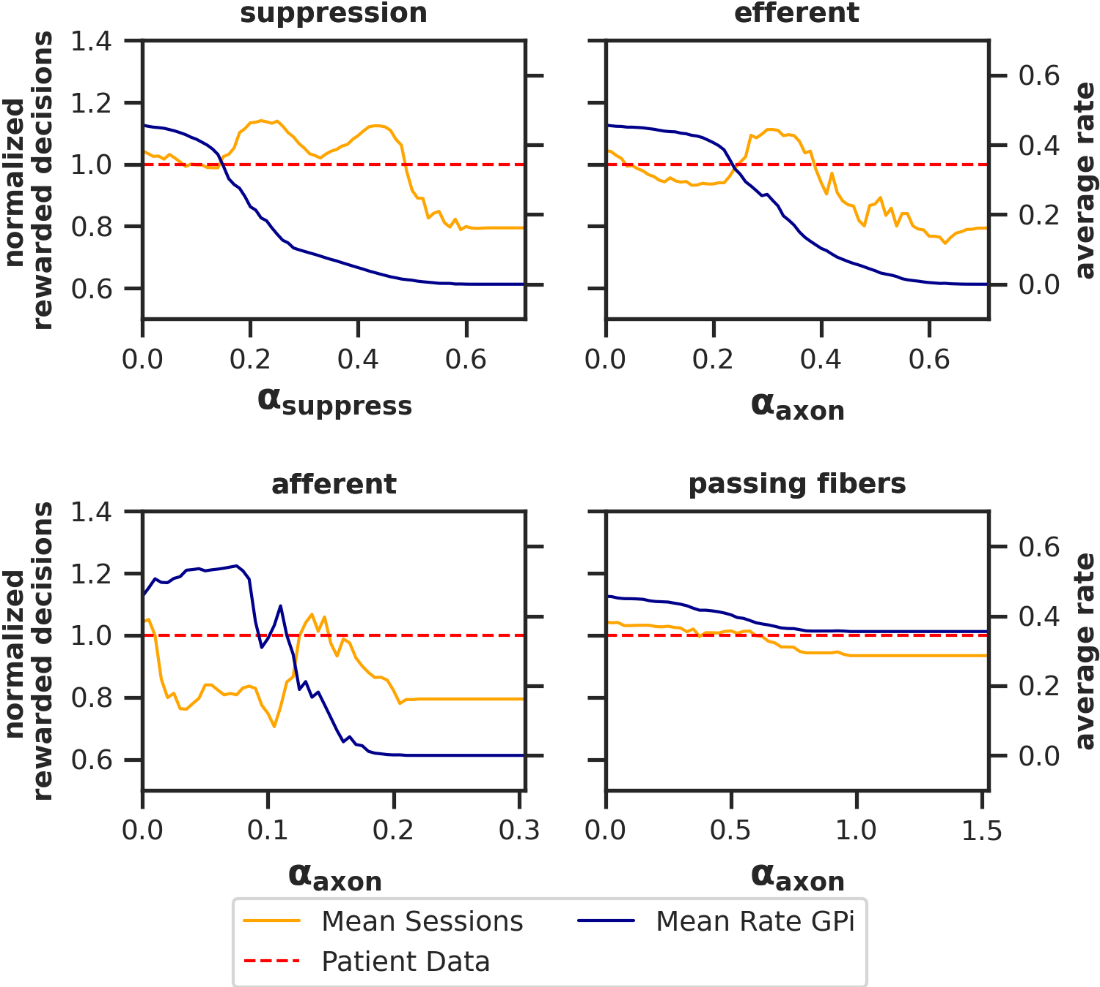


**Figure S6** Related to Figure **8** and Figure **12** of the main article. Normalized number of rewarded decisions averaged over the three sessions of the task for various parameter sets of the simulated DBS variants (orange line) together with the average rate of the GPi population (blue line) obtained from single trials performed by the untrained model. The number of rewarded decisions for each session is normalized by the patients’ data of the corresponding session (dashed red line). The data are averaged over 14 simulations/patients.


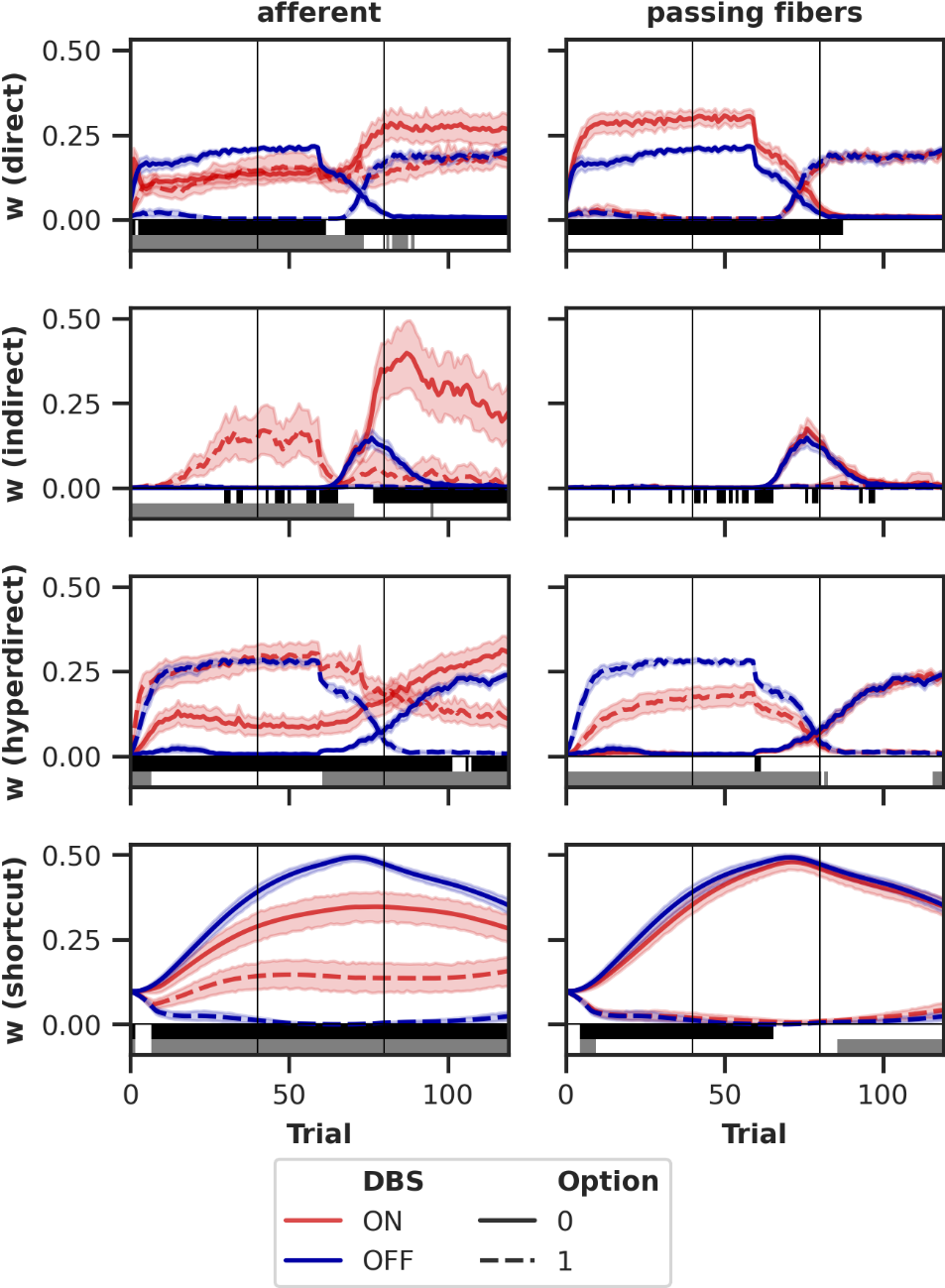


**Figure S7** Related to Figure **9** in the main article. Average calculated weights from the Cor_in_ to the two output neurons in Cor_dec_. Each row displays a different plastic pathway and each column a different DBS variant. The vertical lines indicate the session boundaries. The weights are averaged over 100 simulations (in each DBS condition) the transparent area displays the 95 % confidence interval. Above the x-axis significant differences between DBS ON and OFF are indicated for the output 0 in black and output 1 in gray. P-values were corrected for multiple comparisons using the Benjamini-Hochberg procedure (Benjamini & Hochberg, 1995).


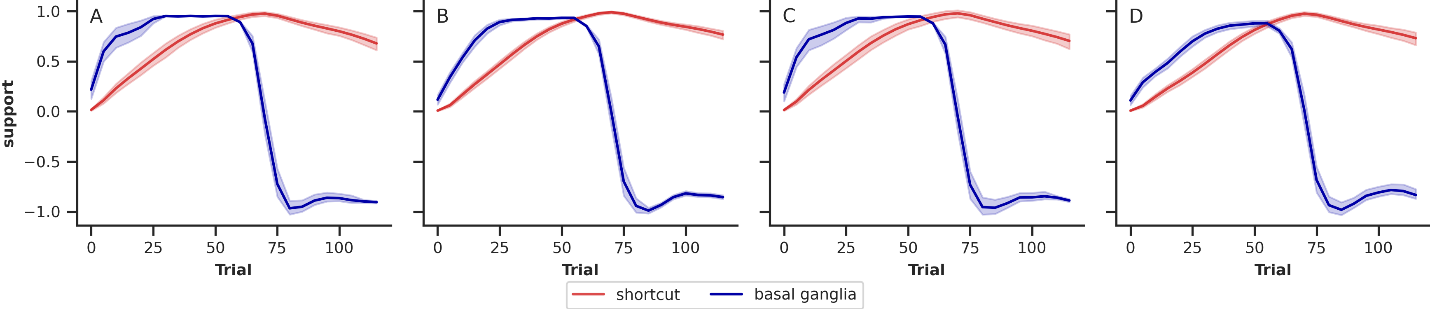


**Figure S8** Related to Figure **10** in the main article. Support of the shortcut and basal ganglia in the thalamus for the initially frequently rewarded option throughout the task in 5-trial bins. A – DBS OFF, B – suppression, C – efferent, D – combined. Notably, basal ganglia support quickly adapts after the reversal at trial 60 while the shortcut support very slowly adapts and still supports the initially frequently rewarded option after the reversal. The lines show the average over 100 simulations with the transparent area representing the 95% confidence interval.


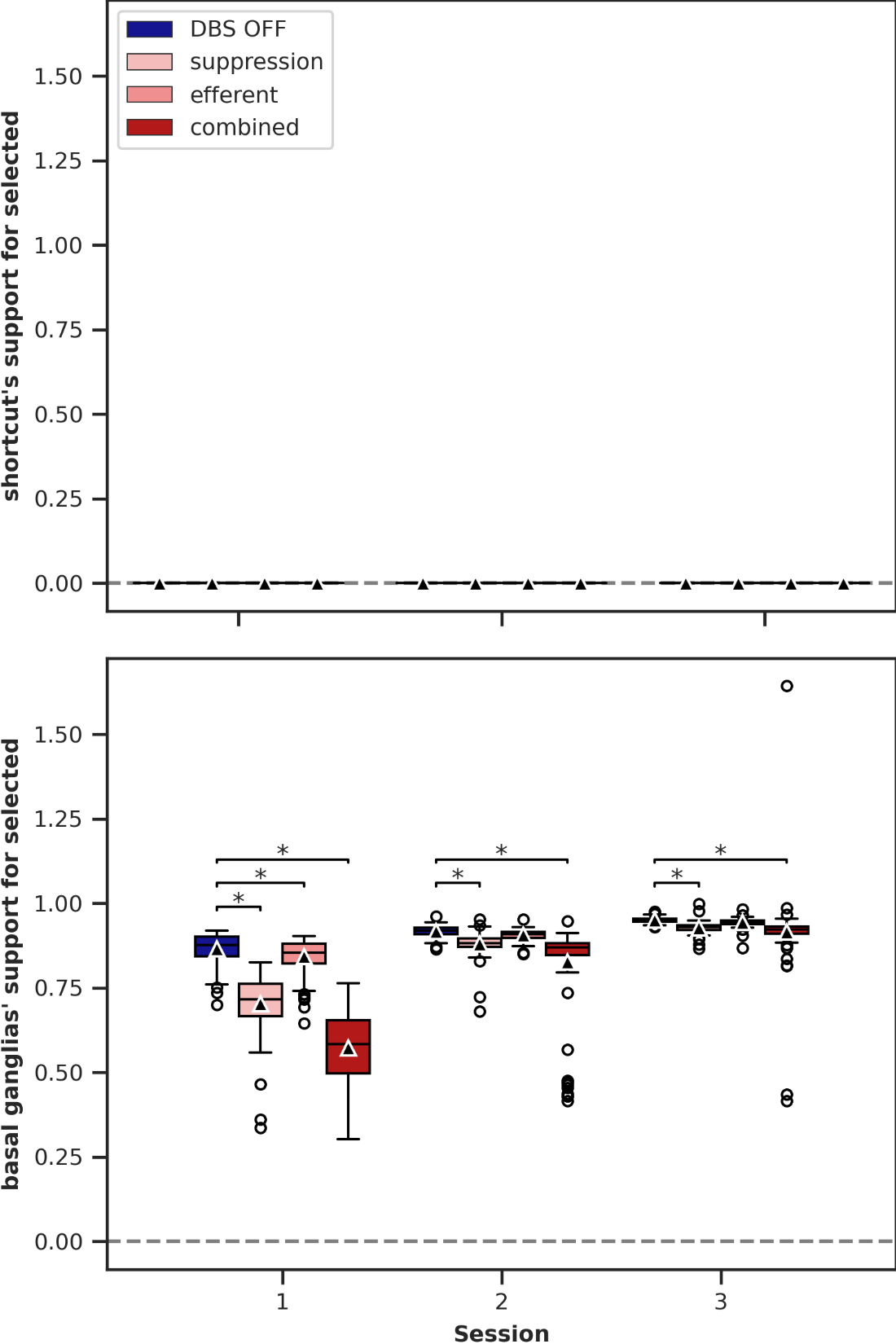


**Figure S9** Related to Figure **10** in the main article. Support of the shortcut and basal ganglia in the thalamus for the selected option for the model with the fixed shortcut. Significant DBS effects (compared to DBS OFF) are annotated. Notably, the decrease of the basal ganglia support induced by DBS is very small in the model with the fixed shortcut. The data of 100 simulations are displayed as boxplots: horizontal line - the median, triangle – the mean, box – the interquartile range (IQR) from the 25^th^ percentile to the 75^th^ percentile, whiskers – extending up to 1.5 times the IQR, circles – outliers outside 1.5 times the IQR.


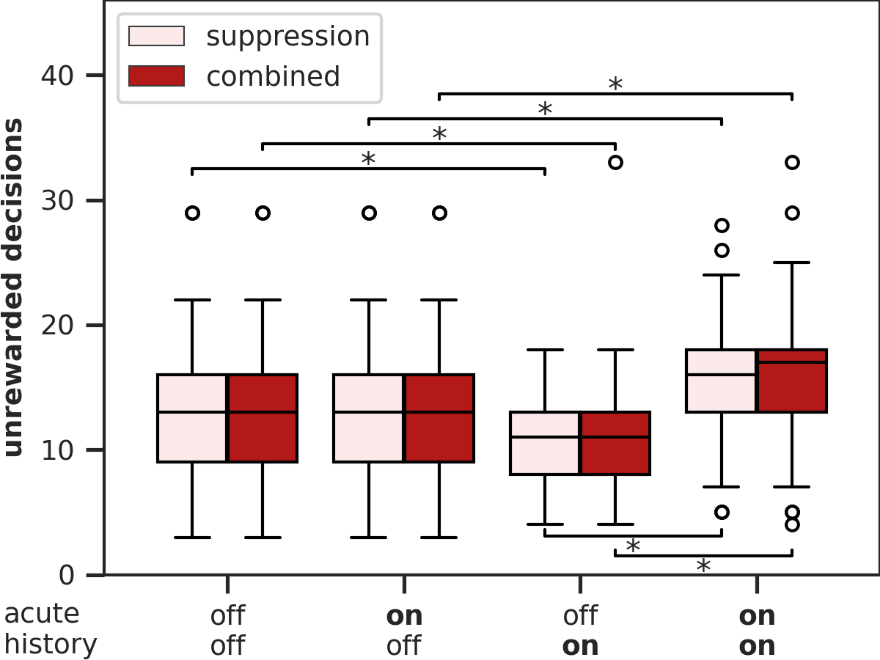


**Figure S10** Related to Figure **11** in the main article. Number of unrewarded decisions in the third session of the task depending on whether DBS is applied during trials (acute) and whether DBS was applied in all trials before (history). Significant effects of acute and history, as identified by post-hoc t-tests, are annotated, for acute below the boxplots and for history above the boxplots. Exclusively applying DBS during trials (acute = on) without applying it in all trials before (history = off) does not lead to any behavioral changes (compared to both off). The DBS variants suppression and combined shown here have almost identical effects on unrewarded decisions. The data of 100 simulations are displayed as boxplots: horizontal line - the median, box – the interquartile range (IQR) from the 25th percentile to the 75th percentile, whiskers – extending up to 1.5 times the IQR, circles – outliers outside 1.5 times the IQR.

## Supplementary Tables

**Table S1** Parameters of dopamine-modulated learning in cortical input projections

| **Presynaptic** | **Postsynaptic** | $\boldsymbol{\tau}_{\boldsymbol{w}}$ | $\boldsymbol{\tau}_{\boldsymbol{\alpha}}$ | $\boldsymbol{\theta}_{\boldsymbol{reg}}$ | $\boldsymbol{\gamma}_{\boldsymbol{burst}}$ | $\boldsymbol{\gamma}_{\boldsymbol{dip}}$ | $\boldsymbol{\beta}$ |
| --- | --- | --- | --- | --- | --- | --- | --- |
| Cor_in_ | StrD1 | 100 | 1 | 0.7 | 4.0 | 0.8 | 1 |
| Cor_in_ | StrD2 | 70 | 1 | 1.0 | 1.2 | 0.8 | -1 |
| Cor_in_ | STN | 70 | 1 | 0.7 | 2.0 | 0.8 | 1 |

Note. $\tau_{w}$ – time constant of the weights, $\tau_{\alpha}$ – time constant of the regularization variable, $\theta_{reg}$ – regularization threshold for postsynaptic membrane potential, $\gamma_{burst}$ learning speed factor for dopamine increases, $\gamma_{dip}$ – learning speed factor for dopamine decreases, $\beta$ factor determining how changes in dopamine modulate learning

**Table S2** Parameters of dopamine-modulated learning in projections within the basal ganglia

| **Presynaptic** | **Postsynaptic** | $\boldsymbol{\tau}_{\boldsymbol{w}}$ | $\boldsymbol{\tau}_{\boldsymbol{\alpha}}$ | $\boldsymbol{\theta}_{\boldsymbol{reg}}$ | $\boldsymbol{\theta}_{\boldsymbol{post}}$ | $\boldsymbol{\gamma}_{\boldsymbol{burst}}$ | $\boldsymbol{\gamma}_{\boldsymbol{dip}}$ | $\boldsymbol{\varphi}$ |
| --- | --- | --- | --- | --- | --- | --- | --- | --- |
| StrD1 | GPi | 50 | 1 | 1.0 | 0.1 | 3.0 | 0.8 | 0.75 |
| StrD2 | GPe | 60 | 1 | 2.0 | 0.15 | 4.0 | 0.8 | 0.10 |
| STN | GPi | 50 | 1 | 1.0 | 0.15 | 2.0 | 0.8 | 1.0 |

Note. $\tau_{w}$ – time constant of the weights, $\tau_{\alpha}$ – time constant of the regularization variable, $\theta_{reg}$ – regularization threshold for postsynaptic membrane potential, $\theta_{post}$ – threshold for postsynaptic rate, $\gamma_{burst}$ – learning speed factor for dopamine increases, $\gamma_{dip}$ – learning speed factor for dopamine decreases, $\varphi$ – learning speed factor depending on the postsynaptic activity

**Table S3** Population parameters of the model from Maith et al. (2021).

| **Population** | **Size** | $\boldsymbol{B}$ | $\boldsymbol{\lambda}$ |
| --- | --- | --- | --- |
| Cor_in_ | 16 | - | - |
| PPN | 1 | - | - |
| SNc | 1 | 0.1 | - |
| StrD1 | 25 | 0.0 | 0.1 |
| StrD2 | 25 | 0.0 | 0.1 |
| STN | 16 | 0.4 | 0.1 |
| GPi | 16 | 2.4 | 0.1 |
| GPe | 16 | 0.4 | 0.1 |
| Thalamus | 16 | 0.35 | 0.1 |
| Cor_dec_ | 16 | 0.0 | 0.05 |
| StrThal | 16 | 0.4 | 0.1 |

Note. Related to **Table 1** of the main article.

**Table S4** Connection types and weights of the projections of the model from Maith et al. (2021).

| **Presynaptic** | **Postsynaptic** | **Connection** | **w** | **target** |
| --- | --- | --- | --- | --- |
| GPe | GPi | one_to_one | 1.5 | inh |
| GPe | STN | - | - | - |
| GPi | GPi | all_to_all | 0.5 | inh |
| GPi | Thalamus | one_to_one | 0.5 | inh |
| Cor_in_ | STN* | all_to_all | (0, 0.3) | exc |
| Cor_in_ | StrD1* | all_to_all | (0, 0.3) | exc |
| Cor_in_ | StrD2* | all_to_all | (0, 0.3) | exc |
| Cor_in_ | Thalamus* | - | - | - |
| Cor_in_ | Thalamus | Gaussian | (0.1, 0.1) | exc |
| PPN | SNc | all_to_all | 1.0 | exc |
| STN | GPe | - | - | - |
| STN | GPi* | all_to_all | (0, 0.05) | exc |
| STN | STN | all_to_all | 0.7 | exc |
| StrD1 | GPi* | all_to_all | (0, 0.05) | inh |
| StrD1 | SNc* | all_to_all | 0.5 | inh |
| StrD1 | StrD1 | all_to_all | 0.7 | inh |
| StrD2 | Gpe* | all_to_all | 0.0 | inh |
| StrD2 | StrD2 | all_to_all | 0.7 | inh |
| StrThal | GPe | one_to_one | 0.3 | inh |
| StrThal | GPi | one_to_one | 0.75 | inh |
| StrThal | StrThal | all_to_all | 1.0 | inh |
| Thalamus | Cor_dec_ | one_to_one | 0.7 | exc |
| Thalamus | StrThal | one_to_one | 0.5 | exc |

Note. Related to **Table 2** of the main article.

**Table S5** Shortcut projection’s plasticity parameters of the model from Maith et al. (2021)

| **Parameter** | **Value** |
| --- | --- |
| $\tau_{w}$ | 15000 |
| $\tau_{\alpha}$ | 1.0 |
| $\theta_{reg}$ | 0.55 |
| $\theta_{post}$ | 0.3 |
| $\theta_{pre}$ | 0.15 |
| $\beta_{reg}$ | 15.0 |

Note. Related to **Table 3** of the main article. $\theta_{pre}$ only exists in the model from Maith et al. (2021). The shortcut projection changed from a cortico-cortical to a cortico-thalamic projection which has the same functionality.

**Table S6** Parameters of dopamine-modulated learning in cortical input projections of the model from Maith et al. (2021)

| **Presynaptic** | **Postsynaptic** | $\boldsymbol{\tau}_{\boldsymbol{w}}$ | $\boldsymbol{\tau}_{\boldsymbol{\alpha}}$ | $\boldsymbol{\theta}_{\boldsymbol{reg}}$ | $\boldsymbol{\theta}_{\boldsymbol{pre}}$ | $\boldsymbol{\gamma}_{\boldsymbol{burst}}$ | $\boldsymbol{\gamma}_{\boldsymbol{dip}}$ | $\boldsymbol{\beta}$ |
| --- | --- | --- | --- | --- | --- | --- | --- | --- |
| Cor_in_ | StrD1 | 75 | 1 | 0.7 | 0.15 | 2.0 | 0.8 | 1 |
| Cor_in_ | StrD2 | 75 | 1 | 1.0 | 0.15 | 6.0 | 0.8 | -1 |
| Cor_in_ | STN | 75 | 1 | 0.7 | 0.15 | 2.0 | 0.8 | 1 |

Note. Related to Table **S1**. $\theta_{pre}$ only exists in the model from Maith et al. (2021).

**Table S7** Parameters of dopamine-modulated learning in projections within the basal ganglia of the model from Maith et al. (2021)

| **Presynaptic** | **Postsynaptic** | $\boldsymbol{\tau}_{\boldsymbol{w}}$ | $\boldsymbol{\tau}_{\boldsymbol{\alpha}}$ | $\boldsymbol{\theta}_{\boldsymbol{reg}}$ | $\boldsymbol{\theta}_{\boldsymbol{post}}$ | $\boldsymbol{\gamma}_{\boldsymbol{burst}}$ | $\boldsymbol{\gamma}_{\boldsymbol{dip}}$ | $\boldsymbol{\varphi}$ |
| --- | --- | --- | --- | --- | --- | --- | --- | --- |
| StrD1 | GPi | 50 | 1 | 0.0 | 0.15 | 2.0 | 0.8 | 1.0 |
| StrD2 | GPe | 50 | 1 | 0.0 | 0.15 | 2.0 | 0.8 | 0.10 |
| STN | GPi | 50 | 1 | 2.6 | -0.15 | 2.0 | 0.8 | 1.0 |

Note. Related to Table **S2**.

**Table S8** Differences in firing rates compared to DBS OFF for all DBS variants

|  |  | **Cor_in** | **StrD1** | **StrD2** | **STN** | **GPi** | **GPe** | **Thalamus** | **Cor_dec** | **StrThal** |
| --- | --- | --- | --- | --- | --- | --- | --- | --- | --- | --- |
| suppression | M | 0,0 | 0,0 | 0,0 | -1,39E-04 | -6,48E-02 | 2,82E-03 | 6,48E-02 | 6,48E-02 | -9,61E-03 |
|  | SD | 0,0 | 0,0 | 0,0 | 8,31E-05 | 3,96E-04 | 1,67E-03 | 3,96E-04 | 3,99E-04 | 5,65E-03 |
| efferent | M | 0,0 | 0,0 | 0,0 | -1,19E-04 | -2,33E-02 | 2,43E-03 | -2,67E-02 | -2,67E-02 | -8,26E-03 |
|  | SD | 0,0 | 0,0 | 0,0 | 2,09E-05 | 1,71E-04 | 3,73E-04 | 1,71E-04 | 1,72E-04 | 1,24E-03 |
| afferent | M | 0,0 | 0,0 | 0,0 | 3,59E-04 | -7,29E-02 | -7,33E-03 | 7,29E-02 | 7,29E-02 | 2,49E-02 |
|  | SD | 0,0 | 0,0 | 0,0 | 1,57E-04 | 2,35E-03 | 3,19E-03 | 2,33E-03 | 2,32E-03 | 1,08E-02 |
| passing fibers | M | 0,0 | 0,0 | 0,0 | -1,38E-02 | 3,62E-03 | -5,38E-03 | -3,62E-03 | -3,62E-03 | -5,30E-04 |
|  | SD | 0,0 | 0,0 | 0,0 | 4,21E-03 | 1,13E-03 | 1,90E-03 | 1,13E-03 | 1,13E-03 | 2,58E-03 |
| combined | M | 0,0 | 0,0 | 0,0 | -1,01E-02 | -7,57E-02 | 3,74E-04 | 4,57E-02 | 4,57E-02 | -1,47E-02 |
|  | SD | 0,0 | 0,0 | 0,0 | 1,82E-03 | 6,89E-04 | 1,80E-03 | 6,89E-04 | 6,89E-04 | 5,95E-03 |

Note. Related to Figure **7** of the main article. M – mean, SD – standard deviation. Data obtained from 100 simulations. Each simulation consisted of a single trial, thus, simulating the untrained model, where the rates were calculated from the time window 2500 – 3000 ms.

**Table S9** Firing rates of the model for DBS OFF and all DBS variants

|  |  | **Cor_in** | **StrD1** | **StrD2** | **STN** | **GPi** | **GPe** | **Thalamus** | **Cor_dec** | **StrThal** |
| --- | --- | --- | --- | --- | --- | --- | --- | --- | --- | --- |
| DBS OFF | M | 1,000 | 0,061 | 0,060 | 0,016 | 0,298 | 0,886 | 0,898 | 0,898 | 0,401 |
|  | SD | 0,000 | 0,007 | 0,007 | 0,006 | 0,003 | 0,004 | 0,003 | 0,003 | 0,011 |
| suppression | M | 1,000 | 0,061 | 0,060 | 0,016 | 0,233 | 0,889 | 0,963 | 0,963 | 0,391 |
|  | SD | 0,000 | 0,007 | 0,007 | 0,006 | 0,003 | 0,003 | 0,003 | 0,003 | 0,006 |
| efferent | M | 1,000 | 0,061 | 0,060 | 0,016 | 0,275 | 0,888 | 0,872 | 0,872 | 0,393 |
|  | SD | 0,000 | 0,007 | 0,007 | 0,006 | 0,003 | 0,004 | 0,003 | 0,003 | 0,010 |
| afferent | M | 1,000 | 0,061 | 0,060 | 0,016 | 0,225 | 0,879 | 0,971 | 0,971 | 0,426 |
|  | SD | 0,000 | 0,007 | 0,007 | 0,006 | 0,003 | 0,003 | 0,003 | 0,003 | 0,001 |
| passing fibers | M | 1,000 | 0,061 | 0,060 | 0,002 | 0,302 | 0,881 | 0,895 | 0,895 | 0,400 |
|  | SD | 0,000 | 0,007 | 0,007 | 0,003 | 0,002 | 0,003 | 0,002 | 0,003 | 0,011 |
| combined | M | 1,000 | 0,061 | 0,060 | 0,006 | 0,222 | 0,886 | 0,944 | 0,944 | 0,386 |
|  | SD | 0,000 | 0,007 | 0,007 | 0,005 | 0,002 | 0,003 | 0,002 | 0,003 | 0,006 |

Note. Related to Figure **7** of the main article. M – mean, SD – standard deviation. Data obtained from 100 simulations. Each simulation consisted of a single trial, thus, simulating the untrained model, where the rates were calculated from the time window 2500 – 3000 ms.

## References

Benjamini, Y., & Hochberg, Y. (1995). Controlling the False Discovery Rate: A Practical and Powerful Approach to Multiple Testing. *Journal of the Royal Statistical Society: Series B (Methodological)*, *57*(1), 289–300.

de A Marcelino, A. L., Gray, O., Al-Fatly, B., Gilmour, W., Douglas Steele, J., Kühn, A. A., & Gilbertson, T. (2023). Pallidal neuromodulation of the explore/exploit trade-off in decision-making. *eLife*, *12*, e79642. https://doi.org/10.7554/eLife.79642

Maith, O., Schwarz, A., & Hamker, F. H. (2021). Optimal attention tuning in a neuro-computational model of the visual cortex–basal ganglia–prefrontal cortex loop. *Neural Networks*, *142*, 534–547. https://doi.org/10.1016/j.neunet.2021.07.008

Schroll, H., Vitay, J., & Hamker, F. H. (2014). Dysfunctional and compensatory synaptic plasticity in Parkinson’s disease. *European Journal of Neuroscience*, *39*(4), 688–702. https://doi.org/10.1111/ejn.12434

Vehtari, A., Gelman, A., & Gabry, J. (2017). Practical Bayesian model evaluation using leave-one-out cross-validation and WAIC. *Statistics and Computing*, *27*(5), 1413–1432. https://doi.org/10.1007/s11222-016-9696-4
